# Liver androgen receptor knockout protects female mice from high fat diet-induced insulin resistance

**DOI:** 10.1038/s44324-026-00133-1

**Published:** 2026-10-01

**Authors:** Md Ahasanur Rahman, Demarrius Young, Taylor Southward, Taylor Lofton, Claire Falzarano, Ella Damaser, Andre Wilson, Damon Ross, Dwight Curry, Jacob Maynard, Jasmine Gray, Jason Lett, Rabia Qutab, Vaisakh Nair, Stanley Andrisse

**Affiliations:** Department of Physiology and Biophysics, Howard University College of Medicine, Washington, DC USA

**Keywords:** Endocrinology, Physiology

## Abstract

High-fat diet (HFD) feeding induces reduced insulin-stimulated AKT phosphorylation with notable sex- and tissue-specific variability, yet the contribution of hepatic androgen receptor (AR) signaling to these effects remains unclear. To investigate the role of hepatic AR in metabolic regulation under dietary stress, liver-specific AR knockout (LivARKO) and wild-type (WT) mice of both sexes were fed control or HFD diets for up to 3 months. HFD impaired insulin-stimulated Akt phosphorylation across liver, skeletal muscle, and white adipose tissue in WT mice. In females, LivARKO preserved hepatic insulin sensitivity and attenuated HFD-induced induction of gluconeogenic genes Pck1 and G6pc, indicating protection from hepatic insulin resistance. This effect was not observed in skeletal muscle or adipose tissue. In contrast, male LivARKO mice showed no protection from HFD-induced insulin resistance and exhibited further upregulation of hepatic Pck1 and G6pc expression compared with WT males. These findings demonstrate a sexually dimorphic role for hepatic AR in regulating insulin signaling and glucose metabolism during HFD feeding. Targeting hepatic AR signaling may offer a sex-specific strategy for mitigating diet-induced metabolic dysfunction and improving metabolic health outcomes.

**Figure:**
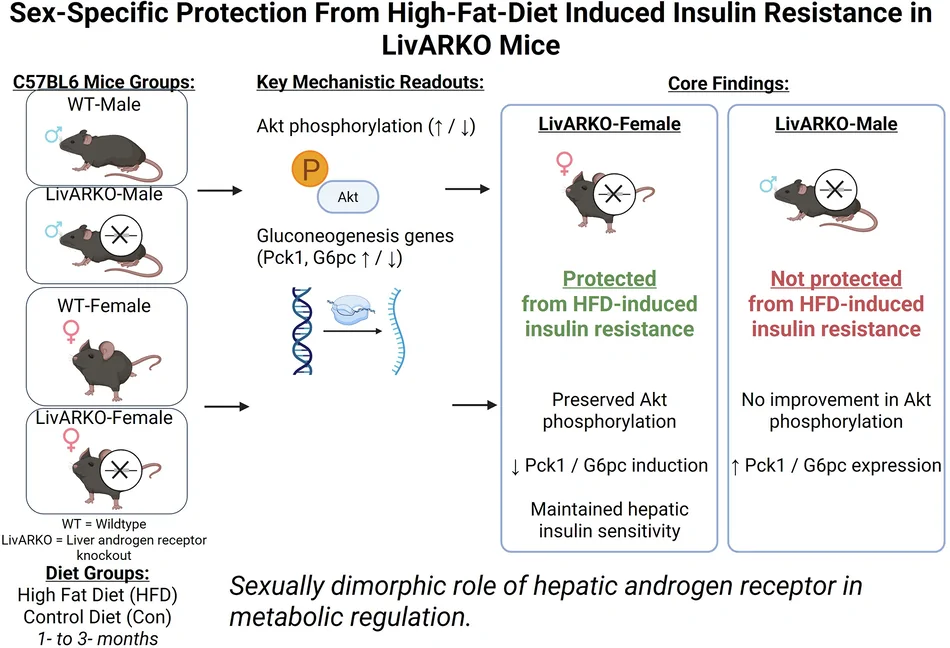

## Introduction

The global rise in obesity and type 2 diabetes mellitus is strongly linked to high-fat diet (HFD) consumption, which promotes insulin resistance in key metabolic tissues including liver, skeletal muscle (SKM), and white adipose tissue (WAT). Although extensively studied in wild-type (WT) male mice, less is known about the sex-specific effects of HFD on insulin signaling pathways. In males, HFD feeding consistently impairs hepatic insulin action, as evidenced by reduced phosphorylation of insulin receptor substrate 1 (IRS1) and AKT^1,2^. By contrast, findings in females are less consistent, with some studies reporting HFD-induced reductions in hepatic phosphorylated AKT (p-AKT) while others observe no change^3–5^. Similarly, HFD-induced insulin resistance in SM and WAT displays notable sex-dependent variability^6–10^. These observations underscore the importance of considering both sex and tissue context when studying diet-induced metabolic dysfunction.

The hepatic androgen receptor (AR) has emerged as a key modulator of glucose and lipid metabolism with distinct, sex-specific roles. In males, hepatic AR signaling appears protective, mitigating steatosis and insulin resistance by promoting fatty acid oxidation and restraining lipogenesis^11^. Conversely, in females, hepatic AR mediates androgen-driven metabolic dysfunction, including whole-body insulin resistance, particularly under conditions of androgen excess such as polycystic ovary syndrome^12^. Recent work further demonstrates that liver androgen receptor knockout (LivARKO) confers protection against HFD-induced glucose intolerance in females but exacerbates hepatic insulin resistance in males^13^.

Based on these findings, we hypothesized that liver AR deletion would differentially affect HFD-induced insulin resistance in males and females. Specifically, we predicted that LivARKO would preserve hepatic insulin sensitivity in females while failing to protect, or even worsening, hepatic insulin resistance in males. To evaluate this hypothesis, we examined insulin signaling and metabolic gene expression in liver, SKM, and WAT of WT and LivARKO mice of both sexes under control and HFD conditions. This approach provides new insight into the sex-specific role of hepatic AR in mediating diet-induced insulin resistance. Importantly, beyond metabolic phenotypes previously reported in this LivARKO model, the present study uniquely defines the tissue-specific and time-dependent insulin signaling responses to hepatic AR deletion by quantifying acute insulin-stimulated AKT phosphorylation across liver, skeletal muscle, and adipose tissue following both short- and prolonged HFD exposure.

## Results

### Verification of LivARKO

To confirm the efficiency and tissue specificity of the liver-specific AR knockout, we measured *Ar* mRNA levels in liver, skeletal muscle, and WAT. As expected, *Ar* expression was significantly reduced in liver tissue of LivARKO mice compared with WT controls (Supplementary Fig. 1), verifying effective deletion. In contrast, *Ar* mRNA levels in skeletal muscle and WAT were unchanged between genotypes (Supplementary Fig. 1), demonstrating that the knockout was restricted to the liver.

### Insulin robustly stimulates Akt phosphorylation in liver, skeletal muscle, and adipose tissue of WT mice; and high-fat diet blunts insulin-induced Akt phosphorylation

Basal Akt phosphorylation (*WT-Con-Bas*) was normalized to 1.0 across all tissues (Supplementary Figs. 2 and 3). Acute insulin stimulation markedly increased p-Akt (Ser473) levels in wild-type mice, ranging from ~3-fold induction in liver and skeletal muscle to ~2.9–3.4-fold in adipose tissue (Supplementary Figs. 2 and 3). These results confirm that insulin robustly activates Akt signaling in metabolic tissues of both male and female mice. In the context of HFD feeding, basal p-Akt levels (*WT-HFD-Bas*) were like or slightly lower than WT-Con-Bas across tissues (Supplementary Figs. 2 and 3). However, insulin-stimulated p-Akt was profoundly reduced in HFD-fed mice. In male liver, insulin increased p-Akt to 3.40 ± 0.15 in control diet-fed mice, but only 0.44 ± 0.16 under HFD (Supplementary Fig. 2A). Similar patterns were observed in male skeletal muscle (3.21 ± 0.17 vs. 0.39 ± 0.12; Supplementary Fig. 2B) and WAT (3.08 ± 0.20 vs. 0.40 ± 0.10; Supplementary Fig. 2C). Female tissues showed a comparable insulin resistance phenotype, with insulin-stimulated p-Akt suppressed by ~80–90% in HFD-fed animals compared to controls (Supplementary Fig. 3A–C). Insulin potently activated Akt phosphorylation in liver, skeletal muscle, and adipose tissue of WT mice, whereas HFD feeding markedly blunted this response in both sexes. These findings demonstrate reduced insulin-stimulated AKT phosphorylation at the level of Akt activation in key metabolic tissues.

### LivARKO females at 1-month HFD prevented the HFD-induced insulin resistance

**Fig. 1:**
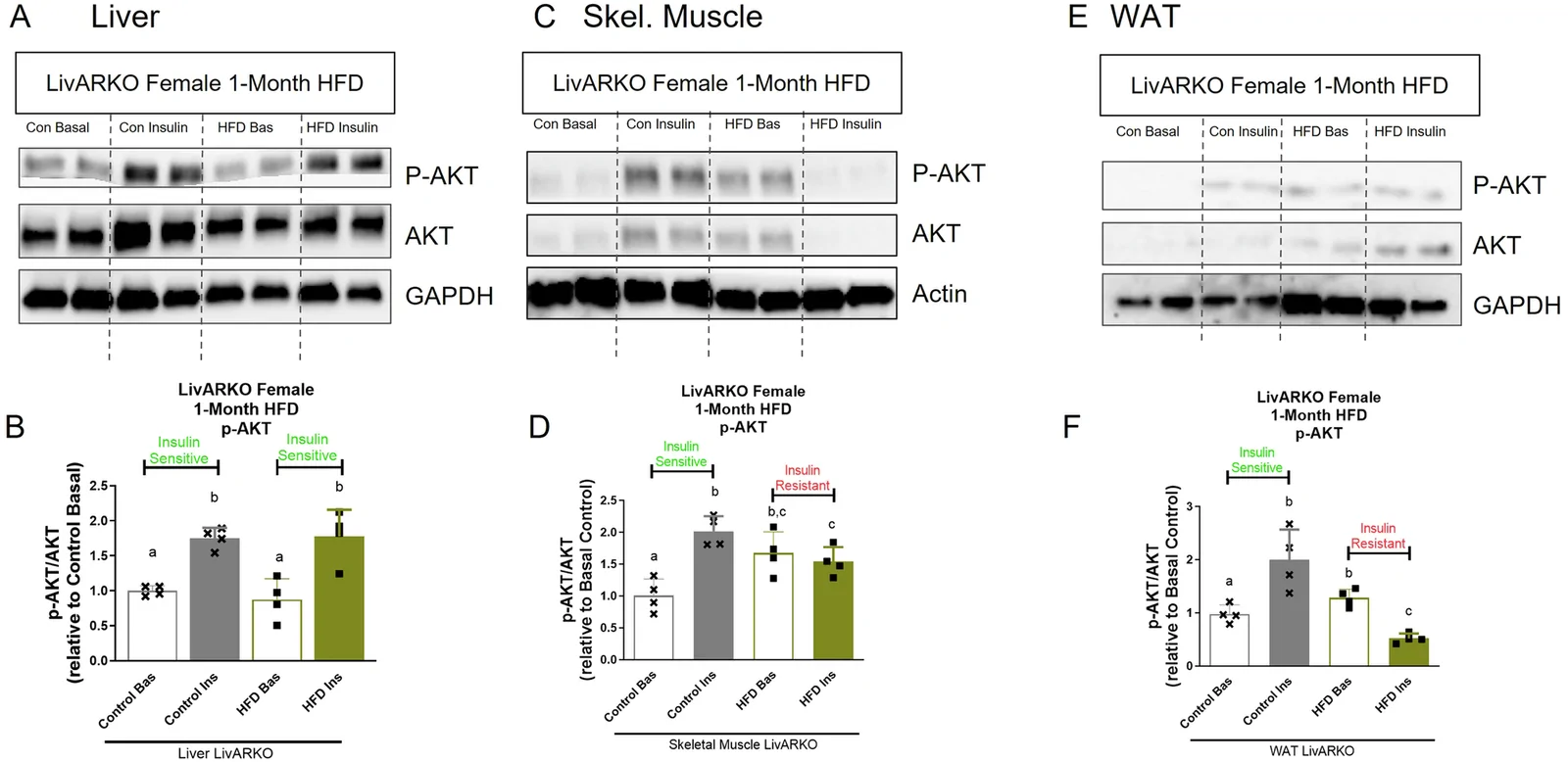
Insulin signaling in female LivARKO mice after 1 month of HFD feeding. Representative Western blots (**A**, **C**, **E**) and densitometric quantification (**B**, **D**, **F**) of p-Akt (Ser473), total Akt, and actin in liver (**A**, **B**), skeletal muscles (SKM) (**C**, **D**), and white adipose tissue (WAT) (**E**, **F**) of liver androgen receptor knockout (LivARKO) female mice fed a control diet (Con) or high-fat diet (HFD) for 1 month, under basal (Bas) or insulin-stimulated (Ins) conditions. Mice were fasted for 6 h prior to tissue collection, and a subset was stimulated with insulin (0.5 U/kg body weight, intraperitoneal injection, 10 min before sacrifice). Insulin significantly increased hepatic p-Akt in both Con and HFD-fed LivARKO mice, indicating preserved hepatic insulin sensitivity under HFD. In contrast, SKM and WAT of LivARKO-HFD females did not respond to insulin, suggesting tissue-specific protection limited to the liver. Mean ± SEM, *n* = 4. Different letters indicate a significant difference at *p* < 0.05.

To evaluate liver insulin action, we assessed p-AKT (Ser473), AKT, and actin (as internal control) protein expression in liver, skeletal muscles, and WAT samples from Control and HFD LivARKO female mice. As seen previously^12^, insulin increased p-AKT in livers of LivARKO mice fed a chow diet. In livers of LivARKO mice fed a control diet for 1-month, insulin increased p-AKT (*p* < 0.001) in comparison to LivARKO mice not given insulin (LivARKO-Control-Basal, Fig. 1A). Interestingly, LivARKO female mice fed a HFD for 1-month, displayed increased insulin-stimulated p-AKT in the liver compared to LivARKO-HFD mice not stimulated with insulin (LivARKO-HFD-Basal, Fig. 1A, B), suggesting that the livers of LivARKO-HFD female mice prevented the HFD-induced insulin resistance seen in WT (Supplementary Fig. 3A). In skeletal muscles of LivARKO female mice fed a control diet for 1-month, insulin increased p-AKT in comparison to LivARKO-Control-Basal female mice (Fig. 1C, D, open gray vs solid gray bars); however, LivARKO-HFD-Basal and LivARKO-HFD-Insulin p-AKT levels remain the same (Fig. 1C, D, open green vs solid green bars), suggesting that LivARKO was unable to protect against HFD-induced skeletal muscle insulin resistance (Supplementary Fig. 3B). Insulin increased p-AKT in WAT of LivARKO female mice fed a control diet for 1-month (LivARKO-Control-Basal compared to LivARKO-Control-Insulin, Fig. 1E, F). Interestingly, LivARKO-HFD-Insulin displayed decreased p-AKT levels compared to LivARKO-HFD-Basal (Fig. 1E, F, open green vs solid green bars), suggesting that LivARKO was unable to protect against HFD-induced WAT insulin resistance (Supplementary Fig. 3C).

### LivARKO males at 1-month HFD exhibited insulin resistance

**Fig. 2:**
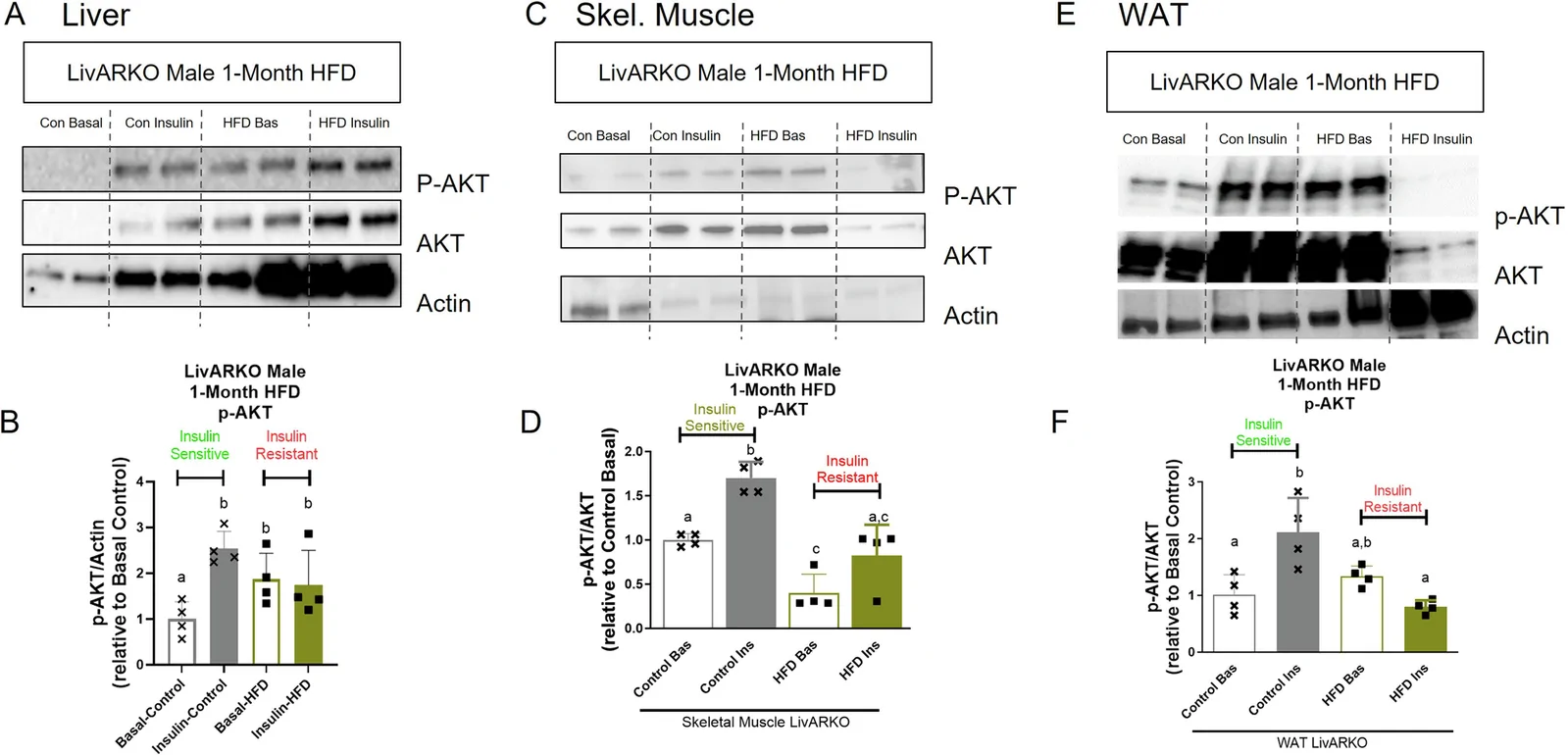
Insulin signaling in male LivARKO mice after 1 month of HFD feeding. Representative Western blots (**A**, **C**, **E**) and quantification (**B**, **D**, **F**) of p-Akt, total Akt, and actin in liver (**A**, **B**), skeletal muscles (SKM) (**C**, **D**), and white adipose tissue (WAT) (**E**, **F**) of liver androgen receptor knockout (LivARKO) males fed a control diet (Con) or a high-fat diet (HFD) for 1 month. Mice were fasted for 6 h prior to tissue collection, and a subset was stimulated with insulin (0.5 U/kg body weight, intraperitoneal injection, 10 min before sacrifice). Insulin robustly stimulated p-Akt in all tissues of Con-fed LivARKO males. However, HFD-fed LivARKO males showed no insulin-induced increase in p-Akt across tissues, indicating lack of hepatic protection. Mean ± SEM, *n* = 4. Different letters indicate a significant difference at *p* < 0.05.

To examine insulin action in LivARKO male mice fed a HFD for 1 month, we assessed p-AKT (Ser473), AKT, and actin protein expression in liver, skeletal muscles, and WAT samples from Control and HFD LivARKO male mice. In the liver (Fig. 2A, B), skeletal muscles (Fig. 2C, D), and WAT (Fig. 2E, F), LivARKO-Control-Insulin male mice displayed significantly increased (*p* < 0.05) p-AKT levels compared to LivARKO-Control-Basal male mice (open gray bars vs solid gray bars). Furthermore, in these three tissues, insulin did not increase p-AKT levels in LivARKO-HFD male mice (Fig. 2, open green bars vs solid green bars), suggesting that LivARKO did protect against the HFD-induced insulin resistance seen in WT male mice (Supplementary Fig. 2). Thus, these data suggest a sexual dimorphism, where LivARKO is protective against HFD-induced hepatic insulin resistance in females, but not in males.

### LivARKO females at 3-month HFD continued to prevent HFD-induced insulin resistance

**Fig. 3:**
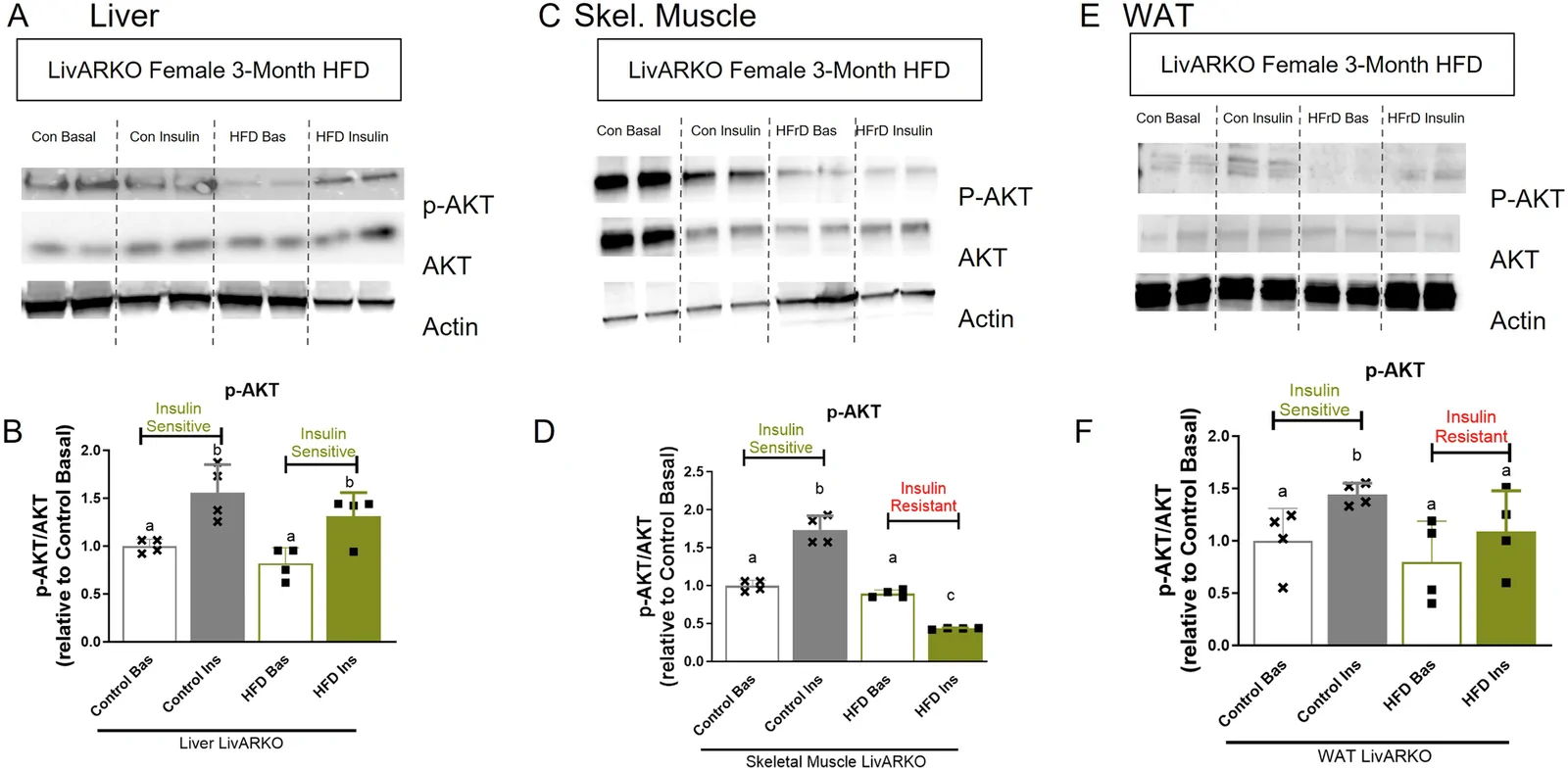
Insulin signaling in female LivARKO mice after 3 months of HFD feeding. Representative Western blots (**A**, **C**, **E**) and densitometric quantification (**B**, **D**, **F**) of p-Akt (Ser473), total Akt, and actin in liver (**A**, **B**), skeletal muscles (SKM) (**C**, **D**), and white adipose tissue (WAT) (**E**, **F**) of liver androgen receptor knockout (LivARKO) female mice fed a control diet (Con) or high-fat diet (HFD) for 3 months, under basal (Bas) or insulin-stimulated (Ins) conditions. Mice were fasted for 6 h prior to tissue collection, and a subset was stimulated with insulin (0.5 U/kg body weight, intraperitoneal injection, 10 min before sacrifice). Insulin significantly increased hepatic p-Akt in Con-fed and HFD-fed LivARKO females, indicating persistent protection of liver insulin signaling after 3 months of HFD. No protection was observed in SKM or WAT, where HFD continued to blunt insulin-stimulated Akt phosphorylation. Mean ± SEM, *n* = 4. Different letters indicate a significant difference at *p* < 0.05.

To evaluate liver insulin action at 3 months of HFD feeding, just as in the 1-month HFD samples, we assessed p-AKT (Ser473), AKT, and actin protein expression in liver, skeletal muscles, and WAT samples from Control and HFD LivARKO female mice. In the liver, after 3 months on the diets, LivARKO-Control-Insulin displayed significantly increased p-AKT (*p* < 0.05) in comparison to LivARKO-Control-Basal mice (Fig. 3A, B, open gray bars vs solid gray bars). Interestingly, LivARKO-HFD-Insulin mice continued to display increased insulin-stimulated p-AKT in the liver at 3 months HFD feeding compared to LivARKO-HFD-Basal mice (Fig. 3A, B, open green bars vs solid green bars), suggesting that LivARKO continued to prevent the HFD-induced insulin resistance seen in WT (Supplementary Fig. 3A). In skeletal muscles of LivARKO female mice fed a control diet for 3 months, insulin increased p-AKT in comparison to LivARKO-Control-Basal female mice (Fig. 3C, D, open gray vs solid gray bars); however, LivARKO-HFD-Basal and LivARKO-HFD-Insulin p-AKT levels were the same (Fig. 3C, D, open green vs solid green bars), suggesting that LivARKO was unable to protect against HFD-induced skeletal muscle insulin resistance (Supplementary Fig. 3B). Insulin increased p-AKT in WAT of LivARKO female mice fed a control diet for 3 months (LivARKO-Control-Basal compared to LivARKO-Control-Insulin, Fig. 3E, F). At 3 months on the diet, LivARKO-HFD-Insulin female mice displayed the same levels of p-AKT compared to LivARKO-HFD-Basal (Fig. 3E, F, open green vs solid green bars), suggesting that LivARKO was unable to protect against HFD-induced WAT insulin resistance (Supplementary Fig. 3C).

### LivARKO males at 3 months HFD continued to exhibit HFD-induced insulin resistance

**Fig. 4:**
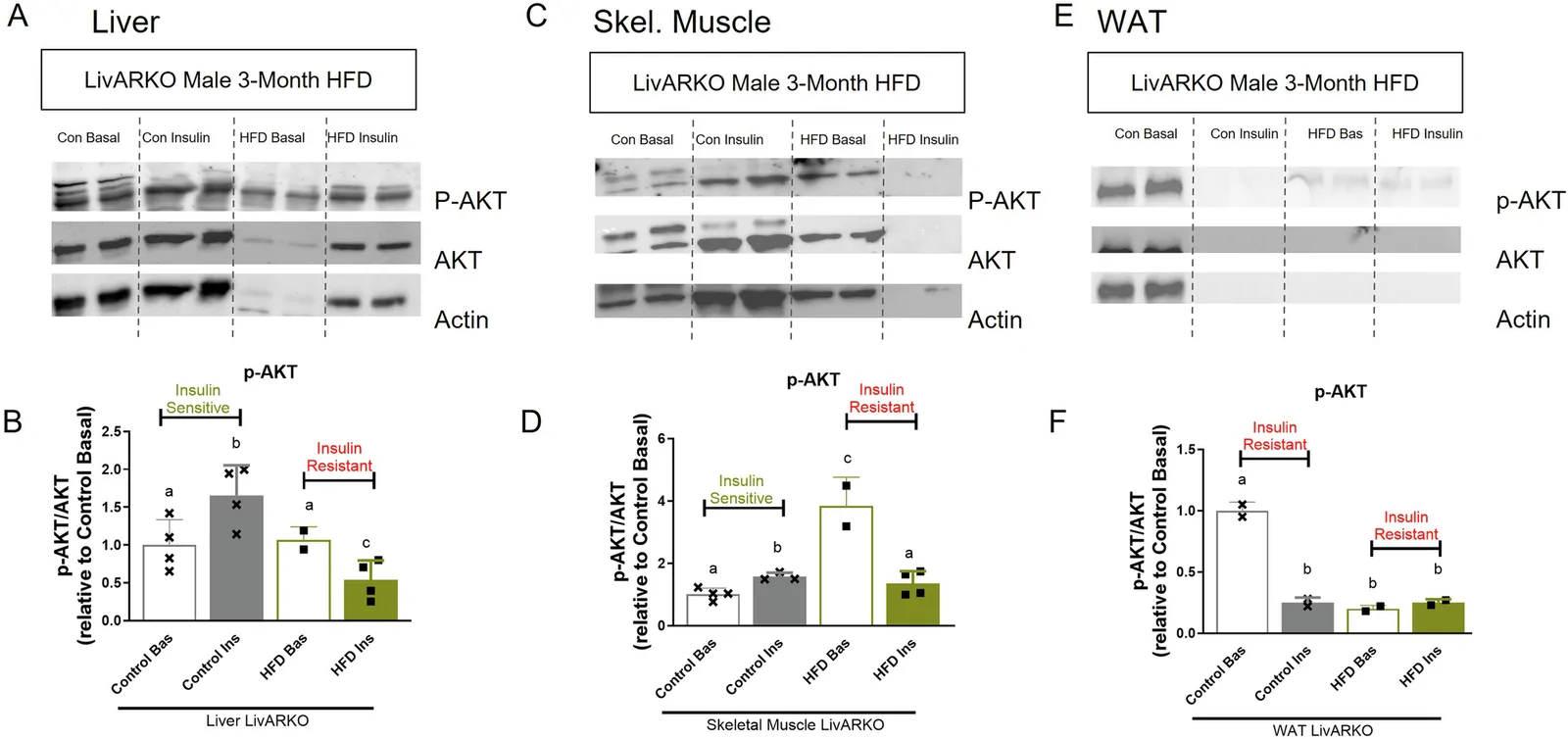
Insulin signaling in male LivARKO mice after 3 months of HFD feeding. Representative Western blots (**A**, **C**, **E**) and quantification (**B**, **D**, **F**) of p-Akt, total Akt, and actin in liver (**A**, **B**), skeletal muscles (SKM) (**C**, **D**), and white adipose tissue (WAT) (**E**, **F**) of liver androgen receptor knockout (LivARKO) males fed a control diet (Con) or a high-fat diet (HFD) for 3 months. Mice were fasted for 6 h prior to tissue collection, and a subset was stimulated with insulin (0.5 U/kg body weight, intraperitoneal injection, 10 min before sacrifice). Con-fed males showed insulin-stimulated increases in p-Akt in liver and SKM, but not WAT. In HFD-fed LivARKO males, insulin failed to stimulate p-Akt in any tissue, demonstrating persistent HFD-induced insulin resistance. Mean ± SEM, *n* = 2–4. Different letters indicate a significant difference at *p* < 0.05.

To explore insulin signaling in LivARKO male mice fed a HFD for 3 months, we investigated p-AKT (Ser473), AKT, and actin protein expression in liver, skeletal muscles, and WAT samples from Control and HFD LivARKO male mice. In the liver (Fig. 4A, B) and skeletal muscles (Fig. 4C, D), but not WAT (Fig. 4E, F), LivARKO-Control-Insulin male mice displayed significantly increased (*p* < 0.05) p-AKT levels compared to LivARKO-Control-Basal male mice (open gray bars vs solid gray bars). Surprisingly, WAT in LivARKO-Control-Insulin male mice displayed lower levels of p-AKT than LivARKO-Control-Basal male mice (Fig. 4E, F). Furthermore, in these three tissues, LivARKO-HFD-Insulin displayed significantly lower p-AKT levels compared to LivARKO-Control-Insulin male mice (Fig. 2, solid gray bars vs solid green bars), suggesting that LivARKO-HFD male mice were experiencing insulin resistance similar to the HFD-induced insulin resistance seen in WT male mice (Supplementary Fig. 2). Thus, these data continue to suggest a sexual dimorphism, where LivARKO is protective against HFD-induced hepatic insulin resistance in females, but not in males.

### Differential expression of gluconeogenic genes suggests a sex-specific divergence

**Fig. 5:**
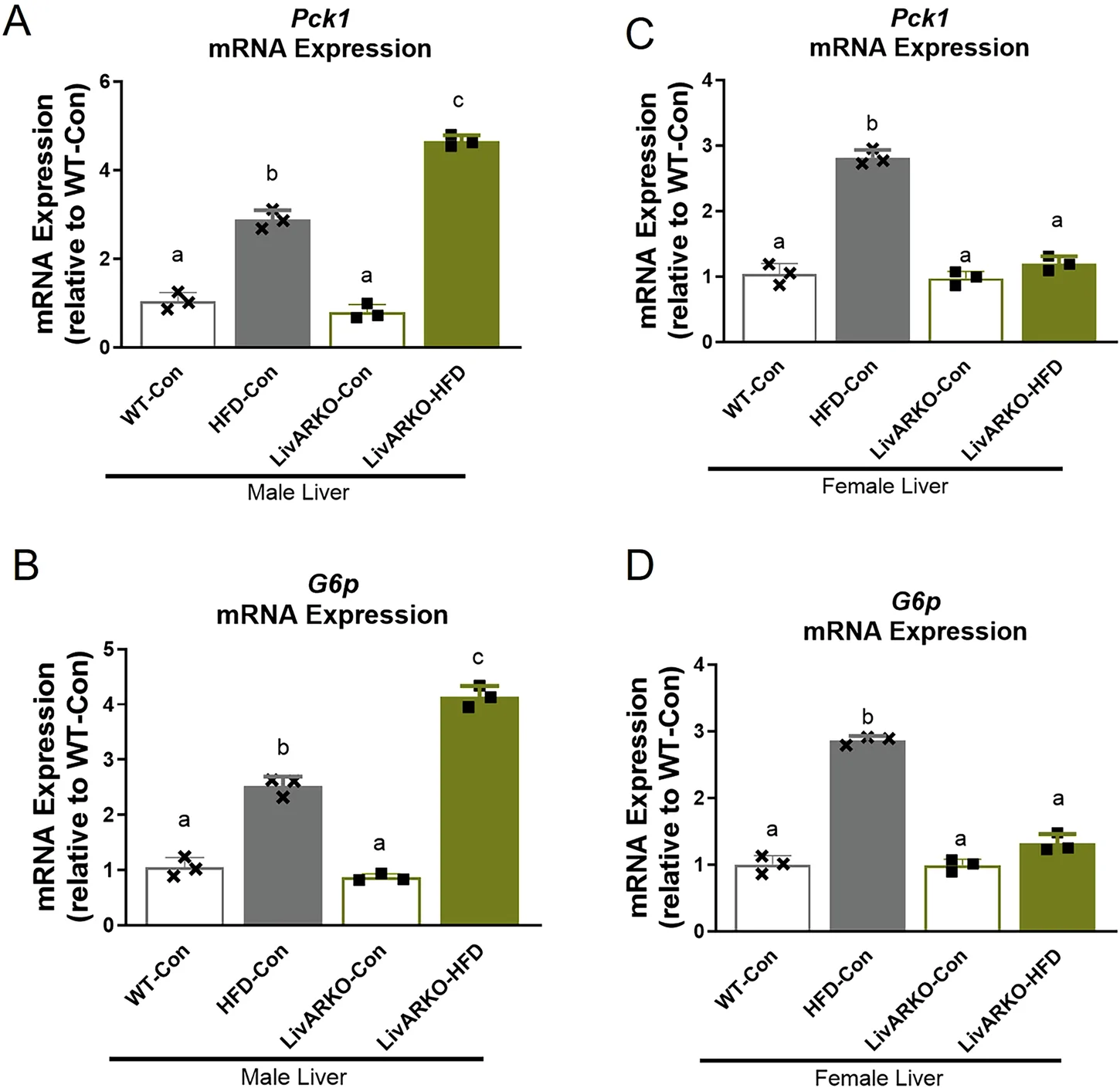
Hepatic gluconeogenic gene expression in WT and LivARKO mice. Relative mRNA levels of *Pck1* (**A**–**C**) and *G6pc* (**B**–**D**) in the liver of male (**A**, **B**) and female (**C**, **D**) wild-type (WT, littermate controls of knockout group) and liver androgen receptor knockout (LivARKO) mice fed a control diet (Con) or a high-fat diet (HFD). In males, HFD markedly increased both genes, and this effect was further exacerbated in LivARKO-HFD mice. In females, HFD also increased *Pck1* and *G6pc* expression in WT, but this induction was blunted in LivARKO-HFD mice. Data are mean ± SEM, *n* = 3 per group. Different letters indicate a significant difference at *p* < 0.05. Statistical analysis was performed using two-way ANOVA with appropriate post hoc testing. Significant main effects of experimental group/diet were detected for all datasets, whereas row factor effects were not significant. Full ANOVA statistics, including interaction analyses, *F* values, and exact *p* values, are provided in the Supplementary Data.

To examine the impact of HFD and liver-specific AR deletion on hepatic gluconeogenic gene expression, we measured mRNA levels of *Pck1* (PEPCK) and *G6pc* (G6Pase) in male and female mice (Fig. 5A–D). In males, HFD feeding markedly increased (*p* < 0.05) both *Pck1* and *G6pc* expression by ~3-fold compared with WT controls (WT-Con: 1.04 ± 0.11 vs. HFD-Con: 2.88 ± 0.12 for *Pck1*; WT-Con: 1.05 ± 0.10 vs. HFD-Con: 2.52 ± 0.10 for *G6pc*; Fig. 5A, B). Strikingly, loss of hepatic AR further exacerbated this response, with LivARKO-HFD males displaying a ~4–5-fold induction (*p* < 0.01) relative to WT-Con (4.65 ± 0.08 for *Pck1*; 4.14 ± 0.11 for *G6pc*; Fig. 5A, B). In contrast, in females, HFD similarly induced *Pck1* and *G6pc* expression compared with WT-Con (2.82 ± 0.06 and 2.86 ± 0.04, respectively; Fig. 5C, D), but this induction was strongly attenuated (*p* < 0.05) in LivARKO-HFD mice (1.50 ± 0.06 for *Pck1*; 1.52 ± 0.07 for *G6pc*; Fig. 5C, D). Basal expression in LivARKO-Con mice was comparable to WT-Con in both sexes. Together, these findings reveal a striking sex-specific divergence: while liver AR deletion in males enhances HFD-induced gluconeogenic gene expression, in females it markedly blunts the HFD effect.

### Previous hormonal findings in the LivARKO model support sex-specific hepatic signaling differences

To provide additional mechanistic context for the sex-specific phenotypes observed in the current study, we incorporated findings from our previously published characterization of this LivARKO model^13^. In that study, circulating estradiol levels were measured in male and female WT and LivARKO mice under control and HFD conditions. Female mice displayed similar serum estradiol levels across all groups examined, and HFD did not significantly alter circulating estrogen levels in male WT or LivARKO mice. Furthermore, female LivARKO mice exhibited no significant differences in hepatic Esr1 mRNA or ERα protein expression under either control diet or HFD conditions. In contrast, male LivARKO-HFD mice displayed reduced hepatic Esr1 mRNA and ERα protein expression, accompanied by increased phosphorylation of ERα at Ser167. Together, these prior findings suggest that the sexually dimorphic metabolic phenotypes observed in the current study are unlikely to be explained solely by differences in circulating estrogen levels and may instead reflect sex-specific hepatic receptor signaling and downstream transcriptional regulation.

## Discussion

In this study, we demonstrate multiple evidence of sexual dimorphism in the role of hepatic androgen receptor (AR) in regulating insulin signaling and gluconeogenic gene expression under conditions of HFD feeding. We confirmed the tissue specificity of our liver AR knockout (LivARKO) model, with *Ar* mRNA expression reduced in liver but unchanged in skeletal muscle or adipose tissue. Using this model, we observed that HFD blunted insulin-stimulated Akt phosphorylation in liver, skeletal muscle, and WAT of wild-type (WT) mice^13^. Importantly, deletion of hepatic AR conferred protection against HFD-induced hepatic insulin resistance in females but not in males, highlighting sex-specific differences in AR function.

Our findings build upon prior studies that established roles for hepatic AR in metabolic regulation. Lin et al.^11^ first showed that global hepatic AR deletion predisposed male mice to steatosis and insulin resistance, whereas subsequent work by Andrisse et al.^12^ demonstrated that hepatic AR mediates androgen-induced metabolic dysfunction in females. More recently, Osei-Ntansah et al.^13^ reported that hepatic AR deletion protects females, but not males, from HFD-induced glucose dysregulation. The present study expands on these reports by directly examining the molecular pathways of insulin signaling at the level of Akt phosphorylation across multiple tissues and by linking these signaling changes to hepatic gluconeogenic gene expression.

While our prior study using this same LivARKO cohort demonstrated that liver-specific androgen receptor deletion improved HFD-induced glucose dysregulation selectively in female mice^13^, the current study substantially extends those findings by defining tissue-specific and temporal insulin signaling responses at the molecular level. Specifically, the present work examined acute insulin-stimulated AKT phosphorylation dynamics across multiple metabolically relevant tissues, including liver, skeletal muscle, and WAT, following both 1-month and 3-month HFD exposure. These analyses revealed that the protective effects of hepatic AR deletion in females were largely restricted to the liver, whereas skeletal muscle and adipose tissue remained insulin resistant under HFD conditions. In contrast, LivARKO males failed to demonstrate preservation of insulin-stimulated AKT signaling across tissues. Additionally, the current study identifies sex-dependent differences in hepatic gluconeogenic gene regulation, with liver AR deletion attenuating HFD-induced *Pck1* and *G6pc* expression in females while exacerbating these responses in males. Collectively, these findings provide new mechanistic insight into how hepatic AR signaling differentially regulates insulin responsiveness and gluconeogenic pathways in males versus females during metabolic stress.

Our findings demonstrated that, in female LivARKO mice, hepatic insulin sensitivity was preserved after 1 and 3 months of HFD feeding, in contrast to WT females, where HFD markedly blunted insulin-induced Akt phosphorylation. These protective effects were tissue-specific, as skeletal muscle and adipose tissue in LivARKO females remained insulin resistant under HFD. Consistent with preserved hepatic insulin signaling, HFD-induced expression of *Pck1* and *G6pc* was attenuated in female LivARKO mice. Together, these findings suggest that loss of hepatic AR in females confers selective protection against HFD-induced hepatic insulin resistance and abnormal gluconeogenic gene regulation.

By contrast, LivARKO males did not show protection. Instead, insulin-stimulated Akt phosphorylation was blunted across tissues under HFD, mirroring WT males. Moreover, hepatic gluconeogenic gene expression was further exacerbated in LivARKO-HFD males compared with WT-HFD males. These results suggest that hepatic AR plays a beneficial role in males, restraining excessive gluconeogenic gene activation under dietary stress, whereas in females, hepatic AR appears detrimental by facilitating insulin resistance and abnormal gene expression. This dichotomy highlights a context- and sex-dependent role of hepatic AR in glucose metabolism.

The mechanisms underlying this sexual dimorphism remain to be fully determined. Possible contributors include differential androgen and estrogen signaling in liver^14^, crosstalk with sex-specific transcriptional programs^15^, and differences in lipid metabolism between sexes^16^. Notably, prior work has shown that androgens exacerbate insulin resistance in females via hepatic AR^12,13,17–19^, while in males hepatic AR appears to support insulin sensitivity through regulation of lipid oxidation pathways^11^. Our findings suggest that targeting hepatic AR may yield opposite outcomes in males versus females, underscoring the need for sex-specific therapeutic strategies.

An additional consideration is the potential contribution of extra-hepatic compensatory endocrine mechanisms to the female-specific metabolic phenotype observed in LivARKO mice. Possible contributors include signaling along the ovary–liver axis^20^, adipose-derived estrogen production^21^, adrenal steroidogenesis^22^, and broader inter-organ endocrine crosstalk^23^ that may differentially influence hepatic insulin sensitivity in females under HFD conditions. While the current study was not designed to directly interrogate these pathways, these mechanisms may represent important contributors to the sexually dimorphic responses observed following hepatic AR deletion and warrant further investigation in future studies.

Additional mechanistic insight may also be derived from our prior hormonal characterization of the LivARKO model^13^. In that study, circulating estradiol levels remained comparable across dietary conditions in both WT and LivARKO females, and HFD did not significantly alter serum estrogen levels in male mice. Interestingly, female LivARKO mice showed no significant changes in hepatic *Esr1* mRNA or ERα protein expression, whereas male LivARKO-HFD mice demonstrated reduced hepatic ERα expression alongside increased p-ERα-S167 signaling. These findings suggest that hepatic estrogen receptor signaling may be differentially regulated between sexes under HFD conditions and could contribute to the divergent metabolic responses observed following hepatic AR deletion. Collectively, these data support the concept that the interaction between androgen receptor and estrogen receptor signaling pathways in the liver may represent an important determinant of sex-specific insulin signaling and gluconeogenic regulation.

Some tissues demonstrated unexpected p-AKT responses following acute insulin stimulation, including reduced or unchanged p-AKT levels under select HFD conditions. These findings likely reflect the complex and context-dependent nature of insulin signaling during chronic metabolic stress rather than simple binary insulin-sensitive versus insulin-resistant states. Chronic HFD exposure may alter receptor sensitivity^1^, downstream negative feedback pathways^24^, phosphatase activity, or basal signaling tone^25^, thereby influencing the magnitude and direction of acute insulin-stimulated AKT responses. Importantly, across most tissues, basal p-AKT levels under HFD conditions were generally similar to or only modestly reduced relative to control diet conditions, whereas insulin-stimulated AKT activation was markedly blunted. These findings suggest that the predominant defect reflects impaired insulin responsiveness rather than uniformly suppressed basal AKT signaling. Thus, HFD exposure appears to primarily disrupt the dynamic capacity of insulin to activate AKT signaling in response to acute stimulation. In some tissues, elevated basal pathway activation may also limit further insulin responsiveness, producing potential ceiling effects^26^. Additionally, acute insulin administration captures dynamic signaling responses at a single time point that may differ from chronic whole-body metabolic adaptation. The sexually dimorphic patterns observed across liver, skeletal muscle, and adipose tissue further suggest that endocrine and tissue-specific regulatory mechanisms influence insulin signaling responses differently in males and females^27^. Collectively, these findings support the concept that hepatic AR deletion interacts with chronic dietary stress in a tissue- and sex-dependent manner to modulate insulin signaling dynamics.

This study has limitations. We focused primarily on Akt phosphorylation as a readout of insulin signaling. To ensure precision in interpretation, we use terminology such as “reduced insulin-stimulated AKT phosphorylation,” “impaired insulin signaling,” and “tissue-specific insulin signaling defects” throughout the manuscript, as AKT phosphorylation represents a molecular readout of insulin action within specific tissues rather than a direct measure of whole-body insulin resistance. Broader metabolic phenotyping of whole-body insulin action in this same LivARKO cohort has been previously reported13, providing complementary context to the signaling-level analyses presented here. Additional analyses of upstream insulin receptor signaling and downstream metabolic endpoints, including glucose production, would provide a more comprehensive understanding of AR function. Moreover, while our study highlights sex differences, we did do an extensive assessment of the role of circulating sex hormones directly, which may be an important modulator of the observed effects. Notably, our previous work (Osei-Ntansah 2024^13^) in this model and the same cohort showed no difference in circulating serum estradiol levels in males or females.

An additional limitation relates to variability in loading control signal quality observed in select Western blot datasets, particularly Fig. 4E (male WAT). Following reviewer feedback, all blots were reanalyzed using improved normalization approaches, and full uncropped blots were included in the Supplementary Data to enhance transparency. While Fig. 4A (liver) and Fig. 4C (skeletal muscle) demonstrated acceptable normalization after reanalysis, variability in AKT and β-actin signal quality persisted in Fig. 4E. Unfortunately, no remaining fresh tissue was available for repeat analysis. Of note, no animals were excluded from analysis. Nevertheless, the overall biological trends remained consistent across tissues and timepoints and are interpreted with appropriate caution.

In summary, we show that deletion of hepatic AR protects female, but not male, mice from HFD-induced hepatic insulin resistance and aberrant gluconeogenic gene expression. These findings reveal a novel sex-dependent role of hepatic AR in metabolic regulation and suggest that strategies targeting hepatic AR should consider sex as a critical biological variable.

## Methods

### Generation of LivARKO mice

Floxed androgen receptor (AR) mice (B6.129S1-Artm2.1Reb/J; The Jackson Laboratory, Bar Harbor, ME, USA) were maintained and bred with Alb-Cre transgenic mice (The Jackson Laboratory) to generate liver-specific androgen receptor knockout (LivARKO) mice (AR^fl/fl^; Alb-Cre^+/−^). LivARKO mice were generated by breeding heterozygous AR^fl/wt^; Alb-Cre^+/−^ females with hemizygous AR^fl/y^; Alb-Cre^−/−^ males. Littermates lacking the Alb-Cre transgene (AR^fl/wt^; Alb-Cre^−/−^ and AR^fl/fl^; Alb-Cre^−/−^) served as littermate controls and are referred to throughout the manuscript as wild-type (WT) mice. Although these mice retain floxed AR alleles, previous characterization demonstrated no significant differences in whole-body glucose metabolism compared with true wild-type animals. Genotyping was performed using genomic DNA isolated from tail biopsies, and primer sequences for AR floxed alleles and Alb-Cre have been previously described^12^.

### Animal ethics, husbandry, randomization, and experimental procedures

All animal experiments were conducted in accordance with the National Institutes of Health Guide for the Care and Use of Laboratory Animals and complied with the ARRIVE 2.0 Essential 10 reporting guidelines. Experimental procedures were reviewed and approved by the Howard University Institutional Animal Care and Use Committee (IACUC-MED-25-02) prior to study initiation.

Mice were housed in a specific pathogen-free animal facility under controlled environmental conditions (22 ± 2 °C) on a 12-h light/12-h dark cycle with unrestricted access to standard chow or experimental diets and water unless otherwise specified. Animals from different experimental groups were housed under identical environmental conditions, and tissue collection and molecular analyses were performed using standardized protocols to minimize batch effects. Animals were monitored daily for health and welfare by trained personnel. No unexpected adverse events occurred, and no humane endpoints were reached before the planned study endpoint.

Male and female mice were randomly assigned to either the control diet or HFD treatment groups at the initiation of dietary intervention. No predefined inclusion or exclusion criteria were established before the study. All animals completing the experimental protocol were included in the analyses. Investigators performing molecular analyses and quantitative image analysis were blinded to treatment group during data analysis.

For insulin signaling experiments, mice were fasted for 6 h before tissue collection. Animals assigned to insulin stimulation received human insulin (0.5 U/kg body weight, intraperitoneal) 10 min before tissue harvest, whereas control animals received an equivalent volume of sterile saline.

At the experimental endpoint, mice were euthanized by cervical dislocation in accordance with AVMA Guidelines for the Euthanasia of Animals and Howard University IACUC-approved protocols. Liver, skeletal muscle, and WAT were rapidly excised, snap frozen in liquid nitrogen, and stored at −80 °C until analysis.

### Genotyping of mouse genomic DNA

Floxed-AR: AAA ATG CCT CCT TTT GAC CA (Forward), AAG ATG ACA GTC CCC ACG AG (Reverse) https://www.jax.org/strain/018450

Alb-Cre: TGC AAA CAT CAC ATG CAC AC (WT Forward), TTG GCC CCT TAC CAT AAC TG (Common), GAA GCA GAA GCT TAG GAA GAT GG (Mutant Forward) https://www.jax.org/strain/003574.

### Experimental design

Male and female C57BL/6 WT mice (as previously described^13^) and LivARKO mice were assigned to one of two diets from 4 to 12 weeks of age:

- Control (Con) diet [Research Diets Inc, RDI D12450J; protein (20%), carbohydrates (70%; corn starch 50%, sucrose-maltodextrin 20%, fructose 0%), fat 10% (lard)].
- HFD [RDI D12492; protein (20%), carbohydrates (20%; corn starch 0%, sucrose-maltodextrin 20%, fructose 0%), fat 60% (lard)].

Research suggests that starting a HFD between 6 and 10 weeks of age produces optimal metabolic outcomes^28^. However, other studies indicate that initiating an HFD as early as 4 weeks of age is suitable for examining metabolic changes during the peripubertal period^29^. To build on our previous findings^12,13,19^ and align with studies using this age range in mice^8,30^, we chose to investigate metabolic disturbances beginning in the peripubertal phase, as it may have relevance to puberty in humans^31^.

### RNA Isolation and qPCR

Frozen tissues were homogenized using a tissue homogenizer (Qiagen, Chatsworth, CA), and total RNA extraction was conducted with TRIzol followed by purification using the miRNeasy Mini Kit (Qiagen) as per the manufacturer’s instructions. Quantitative and qualitative assessments of the RNA were conducted using the BioTek Epoch Microplate Spectrophotometer (Agilent Technologies, Palo Alto, CA), with samples demonstrating RNA integrity >6.5. Subsequently, one microgram of RNA was reverse transcribed using the iScript cDNA synthesis kit (Bio-Rad). Quantitative PCR was then performed utilizing ten nanograms of cDNA on the CFX Duet Touch System (Bio-Rad), employing the iTaq universal SYBR Green Supermix (Bio-Rad). Samples were run in duplicates, and relative gene expression was determined as the mean per group using the ΔΔCt method, normalized to the mean of reference genes (*Actb*). Primer sequences are provided below.

### qRT-PCR Primers: Sense (5′ to 3′) and Antisense (5′ to 3′)

Phosphoenolpyruvate carboxykinase (*Pck1*); Sense AGCATTCAACGCCAGGTTC and Antisense CGAGTCTGTCAGTTCAATACCAA

Glucose 6 phosphatase (*G6p*c); Sense GCTGTGATTGGAGACTGGCTCA and Antisense GTCCAGTCTCACAGGTTACAGG

Androgen receptor (*Ar*); Sense CGGAAGCTGAAGAAACTTGG and Antisense ATGGCTTCCAGGACATTCAG

Actin (*Actb*); Sense CATTGCTGACAGGATGCAGAAGG and Antisense TGCTGGAAGGTGGACAGTGAGG.

### Protein processing and Western blots

Liver, white adipose, and skeletal muscle tissues from male and female mice were harvested following a 6-h fasting period. Ten minutes before euthanasia, mice received an intraperitoneal injection of either saline or insulin (0.5 units/kg BW; Lilly, Indianapolis, IN). Extracted tissues were analyzed for total protein content using the bicinchoninic acid protein assay. Proteins were then prepared for Western blot analysis, including SDS-PAGE separation and transfer to nitrocellulose membranes (Bio-Rad). After blocking, membranes were incubated with primary antibodies against AKT, p-AKT-S473, and actin at varying dilutions (see Table 1). Antibodies were obtained from Santa Cruz Biotechnology and/or Cell Signaling Technologies, as stated in Table 1. Following primary antibody incubation, secondary antibodies (goat anti-mouse IgG or mouse anti-rabbit) were applied at a 1:5000 dilution (Santa Cruz Biotechnology), and protein detection was performed using enhanced chemiluminescence (ChemiDoc imaging, Bio-Rad). Chemiluminescent images were acquired using the ChemiDoc Imaging System (Bio-Rad) with exposure times optimized individually for each target to avoid signal saturation and maintain a linear detection range. Minimal image processing was applied uniformly across the entire blot for brightness and contrast adjustment. Quantification of band intensity was performed using myImage Analysis software (Thermo Fisher Scientific). Phospho-AKT (Ser473) signal was normalized to total AKT, and where appropriate, loading controls (β-actin) were used to verify equal protein loading across samples. Uncropped blots are provided in the Supplementary Data. Densitometric analysis was conducted using myImage Analysis software (Thermo Fisher Scientific), and statistical analysis was performed using two-tailed t-tests in Prism software. This study’s primary outcome measure was insulin-stimulated hepatic AKT phosphorylation.

**Table 1:** Primary antibodies used for Western blot analysis

Peptide/Protein target [kDa]	Antibody ID (RRID)	Name of antibody	Antibody manufacturer, catalog no.	Animal in which antibody was raised; monoclonal or polyclonal	Dilution
Akt1/2/3 (5C10) [62 kDa]	AB_1118808	Akt1/2/3 (5C10)	Santa Cruz Biotechnology, sc-81434	Mouse, Monoclonal	1:1000
p-Akt1/2/3 (Protein kinase B) [62 kDa]	AB_2861344	p-AKT S473 (C-11)	Santa Cruz Biotechnology, sc-514032	Mouse, Monoclonal	1:500
β-Actin [43 kDa]	AB_626632	β-Actin (c4)	Santa Cruz Biotechnology, sc-47778	Mouse, Monoclonal	1:1000
Glyceraldehyde 3-phosphate dehydrogenase [37 kDa]	AB_10847862	GAPDH (g-9)	Santa Cruz Biotechnology, sc-365062	Mouse, Monoclonal	1:1000

The table lists the peptide or protein targets, antibody identifiers (RRID), commercial antibody names, suppliers with catalog numbers, host species and clonality, and the working dilutions used in the study. Monoclonal antibodies were raised in mouse.

### ELISA quantification of Akt phosphorylation

Liver, skeletal muscle (SKM), and epididymal or periovarian WAT were harvested from wild-type (WT) male and female mice maintained on either a control diet or a HFD. Mice were fasted for 6 h prior to tissue collection, and a subset was stimulated with insulin (0.5 U/kg body weight, intraperitoneal injection, 10 min before sacrifice). Tissues were rapidly excised, snap frozen in liquid nitrogen, and stored at –80 °C until processing. For protein extraction, frozen tissues were homogenized in ice-cold Cell Signaling Technology (CST) lysis buffer supplemented with protease and phosphatase inhibitor cocktails (CST #9803). Lysates were cleared by centrifugation (14,000 × *g*, 10 min, 4 °C), and supernatants were collected for ELISA analysis. Protein concentration was determined by BCA assay (Thermo Fisher Scientific).

Phosphorylated Akt (Ser473) and total Akt levels were quantified using the PathScan® Phospho-Akt (Ser473) Sandwich ELISA Kit (#7160, Cell Signaling Technology) and the PathScan® Total Akt Sandwich ELISA Kit (#7146, Cell Signaling Technology), respectively, according to the manufacturer’s instructions. Briefly, 100 µg of total protein per well was loaded in triplicate into antibody-coated 96-well plates. After incubation and washing, captured Akt proteins were detected using a horseradish peroxidase (HRP)-linked secondary antibody and TMB substrate. The reaction was stopped with 2 N sulfuric acid, and absorbance was measured at 450 nm using a microplate reader (BioTek Synergy HT).

For each tissue, relative levels of phosphorylated Akt were normalized to the mean value of the WT control (WT-Con), which was set to 1.0. Corresponding total Akt measurements were used to confirm equal protein loading and Akt expression across conditions. Data were collected from *n* = 3 biological replicates per group. The four experimental groups included:

1. Wild-type control (WT-Con),
2. Wild-type insulin-stimulated (WT-Ins),
3. High-fat diet control (HFD-Con), and
4. High-fat diet insulin-stimulated (HFD-Ins).

Normalized group means and standard error of the mean (SEM) were calculated for each dataset. The resulting six datasets represent relative phospho-Akt levels in male and female liver, skeletal muscle, and WAT.

### Power analysis

The study utilized the “resource equation” method and sought guidance from a biostatistician to ensure appropriate statistical power. All comparisons were adequately powered, although comparisons involving both male and female subjects were slightly overpowered due to the necessity for a larger sample size when comparing only one gender.

### Statistical analysis

Group comparisons were conducted using a 2-way ANOVA, with data presented as mean ± SEM. Pearson correlation was employed to examine relationships between variables, while Fisher’s exact test was used to compare sex proportions. Two-way ANOVA was used to compare groups for continuous variables. Statistical significance was defined as *P* < 0.05. Data were assessed for normality and homogeneity of variance before parametric analyses. No violations requiring alternative statistical methods were identified.

For interpretive purposes throughout the manuscript and figures, “insulin sensitive” refers to conditions in which insulin stimulation significantly increased p-AKT relative to basal conditions, whereas “insulin resistant” refers to conditions in which insulin failed to increase or reduced p-AKT signaling relative to basal levels.

## Supplementary information

Supplementary Information

## Data Availability

The datasets generated and/or analyzed during the current study are not publicly available because they contain primary research data that are part of ongoing investigations but are available from the corresponding author upon reasonable request.

## References

[1] Liu, Z. et al. High-fat diet induces hepatic insulin resistance and impairment of synaptic plasticity. *PLoS ONE* **10**, e0128274, 10.1371/journal.pone.0128274 (2015). PMC4449222 26023930

[2] Hillian, A. D. et al. Mice lacking C1q are protected from high fat diet-induced hepatic insulin resistance and impaired glucose homeostasis. *J. Biol. Chem.* **288**, 22565–22575, 10.1074/jbc.M113.465674 (2013). PMC3829343 23788643

[3] Pettersson, U. S., Walden, T. B., Carlsson, P. O., Jansson, L. & Phillipson, M. Female mice are protected against high-fat diet induced metabolic syndrome and increase the regulatory T cell population in adipose tissue. *PLoS ONE* **7**, e46057, 10.1371/journal.pone.0046057 (2012). PMC3458106 23049932

[4] Nishio, N. & Isobe, K. GADD34-deficient mice develop obesity, nonalcoholic fatty liver disease, hepatic carcinoma and insulin resistance. *Sci. Rep.* **5**, 13519. 10.1038/srep13519 (2015). PMC4551985 26316333

[5] Lai, H. et al. High-fat diet induces significant metabolic disorders in a mouse model of polycystic ovary syndrome. *Biol. Reprod.* **91**, 127, 10.1095/biolreprod.114.120063 (2014). 25100714

[6] White, A. T. et al. High-fat diet-induced impairment of skeletal muscle insulin sensitivity is not prevented by SIRT1 overexpression. *Am. J. Physiol. Endocrinol. Metab.* **307**, E764–E772, 10.1152/ajpendo.00001.2014 (2014). PMC4216952 25159328

[7] Li, N. et al. Aerobic exercise prevents chronic inflammation and insulin resistance in skeletal muscle of high-fat diet mice. *Nutrients* **14**, 10.3390/nu14183730 (2022). PMC9503887 36145106

[8] Bubak, M. P. et al. 17alpha-estradiol alleviates high-fat diet-induced inflammatory and metabolic dysfunction in skeletal muscle of male and female mice. *Am. J. Physiol. Endocrinol. Metab* **326**, E226–E244 (2024). 10.1152/ajpendo.00215.2023 PMC11193529 38197793

[9] Rudnicki, M. et al. Female mice have higher angiogenesis in perigonadal adipose tissue than males in response to high-fat diet. *Front. Physiol.* **9**, 1452, 10.3389/fphys.2018.01452 (2018). PMC6206240 30405427

[10] He, M. Q. et al. High-fat diet-induced adipose tissue expansion occurs prior to insulin resistance in C57BL/6J mice. *Chronic Dis. Transl. Med.* **6**, 198–207, 10.1016/j.cdtm.2020.06.003 (2020). PMC7451745 32885155

[11] Lin, H. Y. et al. Increased hepatic steatosis and insulin resistance in mice lacking hepatic androgen receptor. *Hepatology* **47**, 1924–1935, 10.1002/hep.22252 (2008). 18449947

[12] Andrisse, S. et al. Androgen-induced insulin resistance is ameliorated by deletion of hepatic androgen receptor in females. *FASEB J.* **35**, e21921. 10.1096/fj.202100961R (2021). PMC9097557 34547140

[13] Osei-Ntansah, A. et al. Liver androgen receptor knockout improved high-fat diet induced glucose dysregulation in female mice but not male mice. *J. Endocr. Soc.* **8**, bvae021. 10.1210/jendso/bvae021 (2024). PMC10904101 38425436

[14] Zheng, D., Wang, X., Antonson, P., Gustafsson, J. A. & Li, Z. Genomics of sex hormone receptor signaling in hepatic sexual dimorphism. *Mol. Cell. Endocrinol.* **471**, 33–41, 10.1016/j.mce.2017.05.025 (2018). PMC5702598 28554805

[15] Burke, M. et al. Sexual dimorphism during integrative endocrine and immune responses to ionizing radiation in mice. *Sci. Rep.* **14**, 7334. 10.1038/s41598-023-33629-7 (2024). PMC10897391 38409284

[16] Varlamov, O., Bethea, C. L. & Roberts, C. T. Jr. Sex-specific differences in lipid and glucose metabolism. *Front Endocrinol.* **5**, 241, 10.3389/fendo.2014.00241 (2014). PMC4298229 25646091

[17] Andrisse, S., Billings, K., Xue, P. & Wu, S. Insulin signaling displayed a differential tissue-specific response to low-dose dihydrotestosterone in female mice. *Am. J. Physiol. Endocrinol. Metab.* **314**, E353–E365, 10.1152/ajpendo.00195.2017 (2018). PMC5966754 29351485

[18] Andrisse, S. et al. Low-dose dihydrotestosterone drives metabolic dysfunction via cytosolic and nuclear hepatic androgen receptor mechanisms. *Endocrinology* **158**, 531–544, 10.1210/en.2016-1553 (2017). PMC5460775 27967242

[19] Seidu, T. et al. DHT causes liver steatosis via transcriptional regulation of SCAP in normal weight female mice. *J. Endocrinol.* **250**, 49–65, 10.1530/JOE-21-0040 (2021). PMC8240729 34060475

[20] WeiskiRchen, R. & Lonardo, A. The ovary-liver axis: molecular science and epidemiology. *Int. J. Mol. Sci.* **26**, 10.3390/ijms26136382 (2025). PMC12250148 40650160

[21] Lee, A. A. & Den Hartigh, L. J. Metabolic impact of endogenously produced estrogens by adipose tissue in females and males across the lifespan. *Front. Endocrinol.* **16**, 1682231. 10.3389/fendo.2025.1682231 (2025). PMC12575157 41180177

[22] Kater, C. E., Giorgi, R. B. & Costa-Barbosa, F. A. Classic and current concepts in adrenal steroidogenesis: a reappraisal. *Arch. Endocrinol. Metab.* **66**, 77–87, 10.20945/2359-3997000000438 (2022). PMC9991025 35263051

[23] Che, H. et al. Organ cross-talk: molecular mechanisms, biological functions, and therapeutic interventions for diseases. *Signal Transduct. Target. Ther.* **11**, 8, 10.1038/s41392-025-02329-1 (2026). PMC12769719 41491917

[24] Petersen, M. C. & Shulman, G. I. Mechanisms of insulin action and insulin resistance. *Physiol. Rev.* **98**, 2133–2223, 10.1152/physrev.00063.2017 (2018). PMC6170977 30067154

[25] Tramunt, B. et al. Sex differences in metabolic regulation and diabetes susceptibility. *Diabetologia* **63**, 453–461, 10.1007/s00125-019-05040-3 (2020). PMC6997275 31754750

[26] Li, M. et al. Trends in insulin resistance: insights into mechanisms and therapeutic strategy. *Signal Transduct. Target. Ther.* **7**, 216, 10.1038/s41392-022-01073-0 (2022). PMC9259665 35794109

[27] Ortiz-Huidobro, R. I., Velasco, M., Larque, C., Escalona, R. & Hiriart, M. Molecular insulin actions are sexually dimorphic in lipid metabolism. *Front. Endocrinol.* **12**, 690484. 10.3389/fendo.2021.690484 (2021). PMC8251559 34220716

[28] Glavas, M. M. et al. Developmental timing of high-fat diet exposure impacts glucose homeostasis in mice in a sex-specific manner. *Diabetes* **70**, 2771–2784, 10.2337/db21-0310 (2021). PMC8660987 34544729

[29] Cordoba-Chacon, J. et al. Peripubertal-onset but not adult-onset obesity increases IGF-I and drives development of lean mass, which may lessen the metabolic impairment in adult obesity. *Am. J. Physiol. Endocrinol. Metab.* **303**, E1151–E1157, 10.1152/ajpendo.00340.2012 (2012). PMC3774069 22932784

[30] Yang, C. et al. Effects of prolonged high-fat diet consumption starting at different ages on behavioral parameters and hippocampal neuroplasticity in male mice. *J. Integr. Neurosci.* **22**, 16, 10.31083/j.jin2201016 (2023). 36722241

[31] Burt Solorzano, C. M. & McCartney, C. R. Obesity and the pubertal transition in girls and boys. *Reproduction* **140**, 399–410, 10.1530/REP-10-0119 (2010). PMC2931339 20802107

