# Supplementary material for: Liver androgen receptor knockout protects female mice from high fat diet-induced insulin resistance
Source: NPJ Metab Health Dis. 2026 Oct 1;4:42. doi: 10.1038/s44324-026-00133-1 (PMC13631308; doi:10.1038/s44324-026-00133-1)
Supplement: Supplementary file 1 [file 44324_2026_133_MOESM1_ESM.pdf]

Supplemental Data

Supplemental Figure 1: Verification of LivARKO

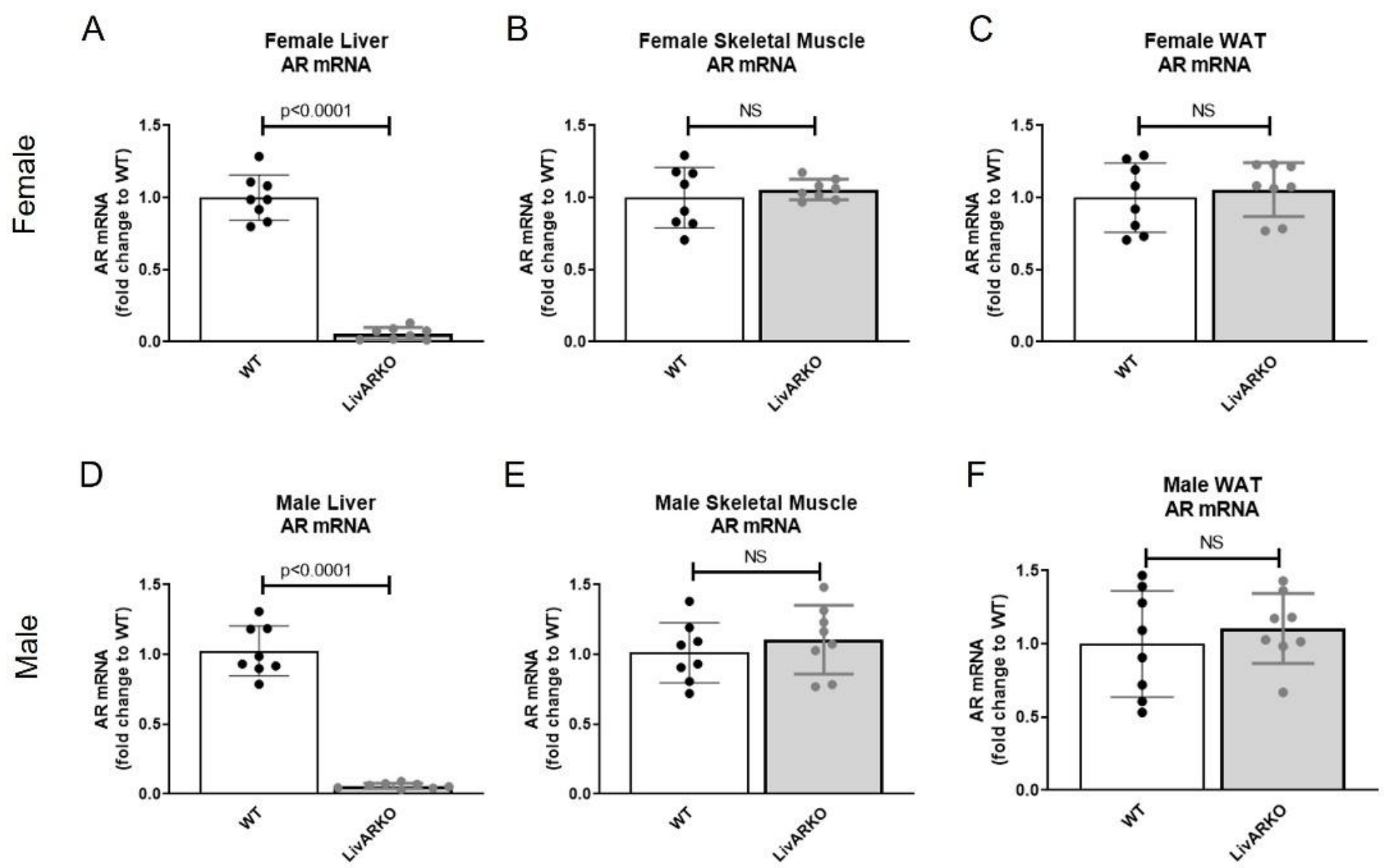

Suppl. Fig. 2 WT-Male p-AKT/AKT

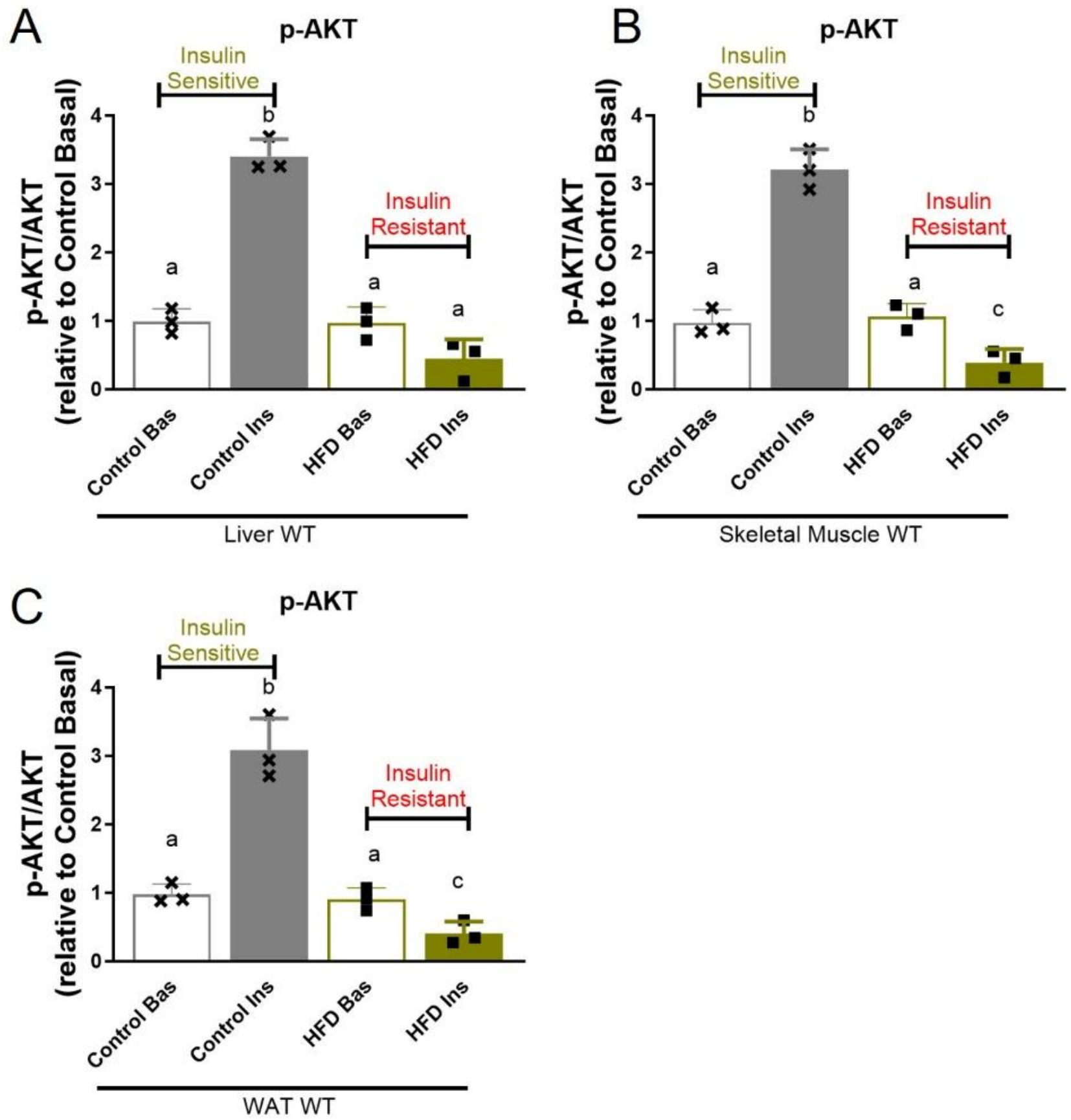

Suppl. Fig. 3 WT-Female p-AKT/AKT

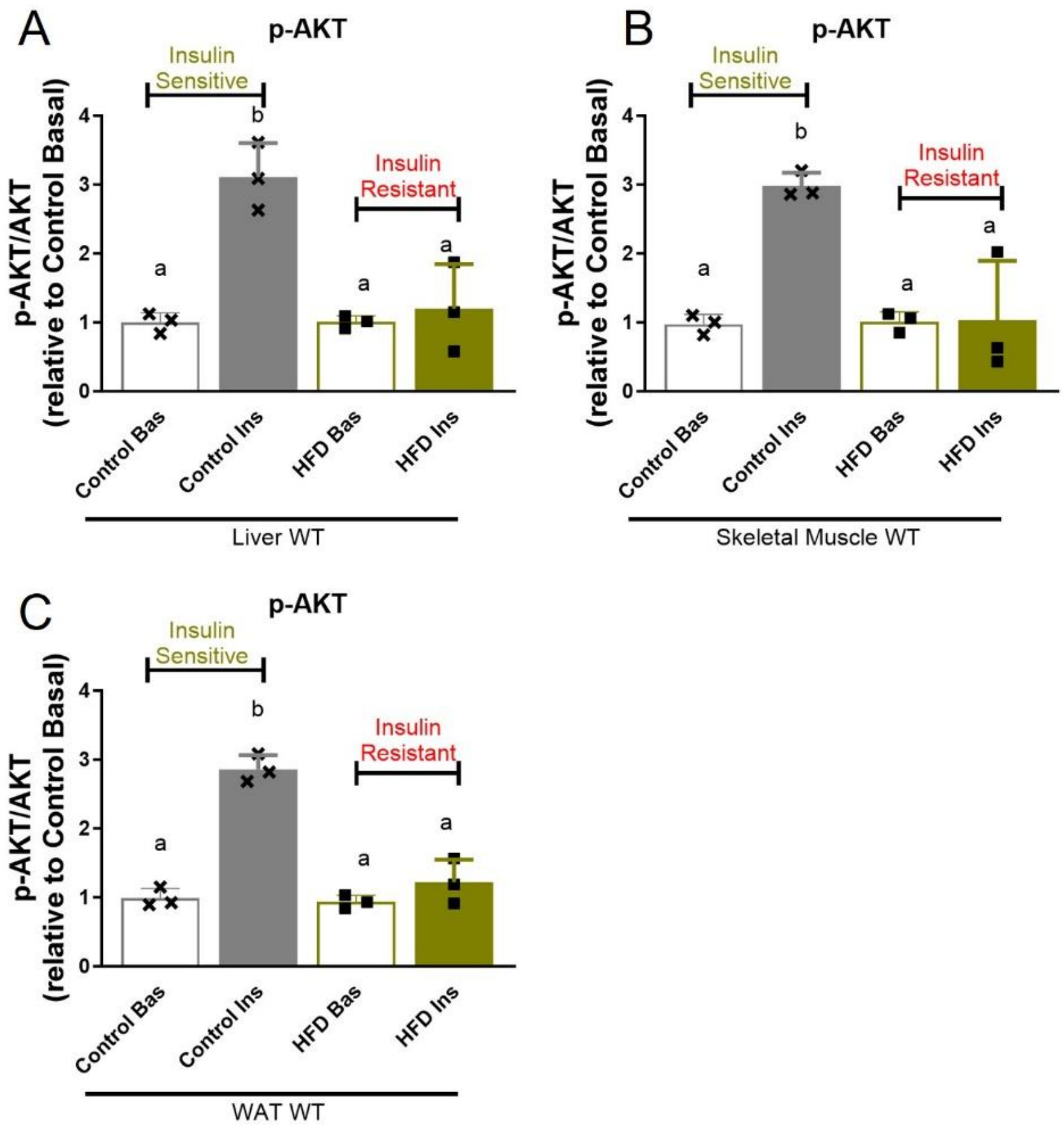

Raw Data

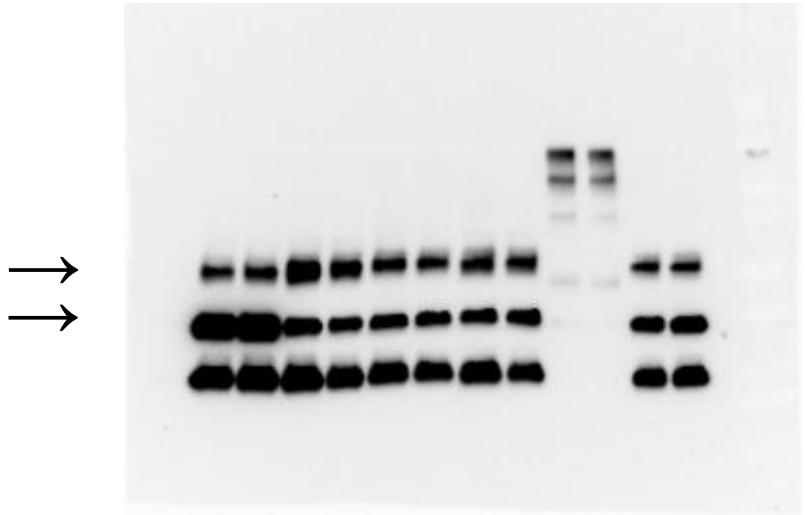

Fig 1A: Liv LivARKO Fem 1-Month HFD (AKT, Actin)

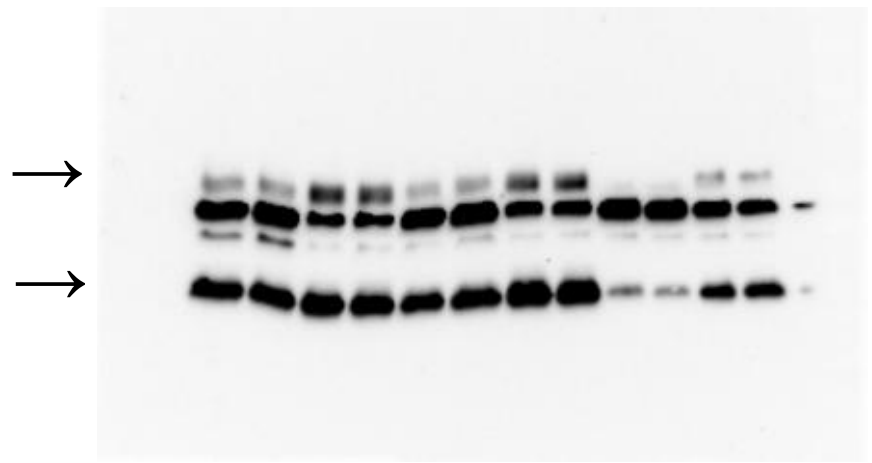

Fig 1A: Liv LivARKO Fem 1-Month HFD (p-AKT, GAPDH)

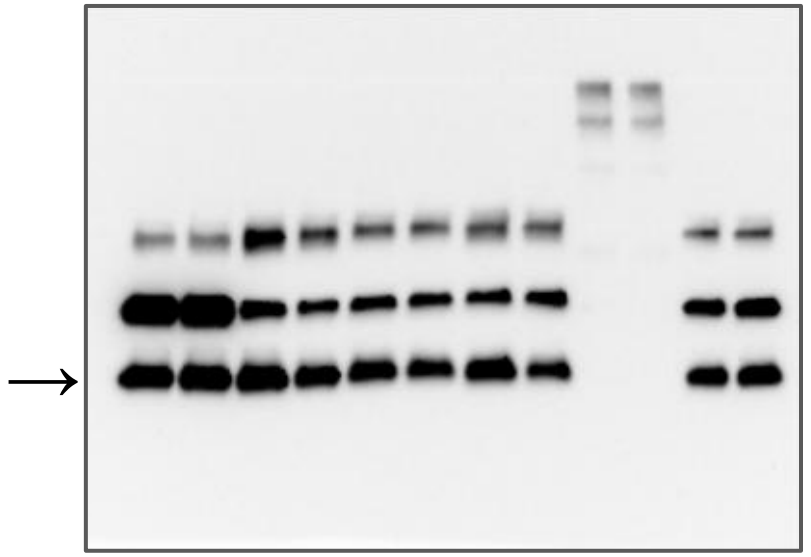

Fig 1A: Liv LivARKO Fem 1-Month HFD (GAPDH)

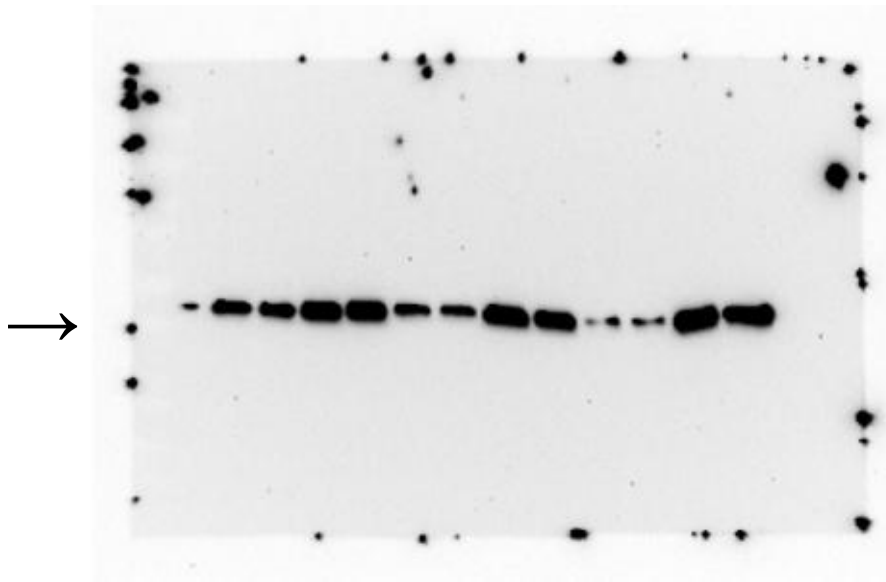

Fig 1A: Liv LivARKO Fem 1-Month HFD ((Actin)

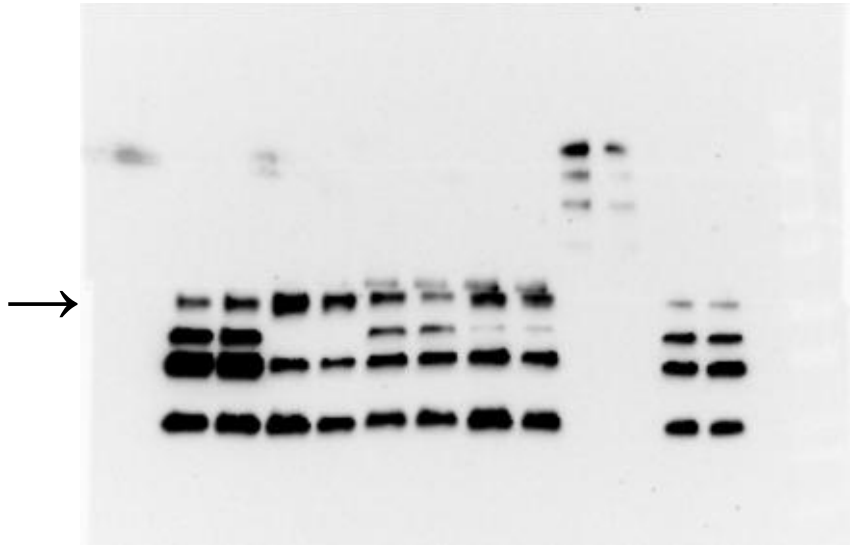

Fig 1A: Liv LivARKO Fem 1-Month HFD ((p-AKT)

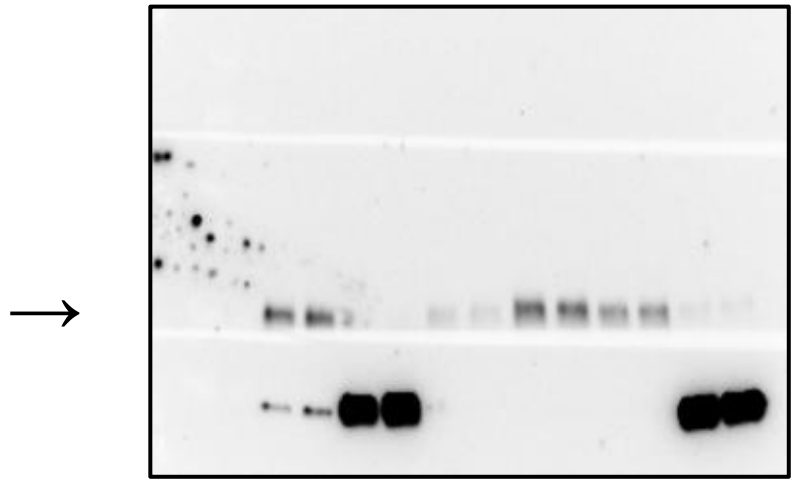

Fig 1C: SM LivARKO Fem 1-Month HFD (p-AKT)

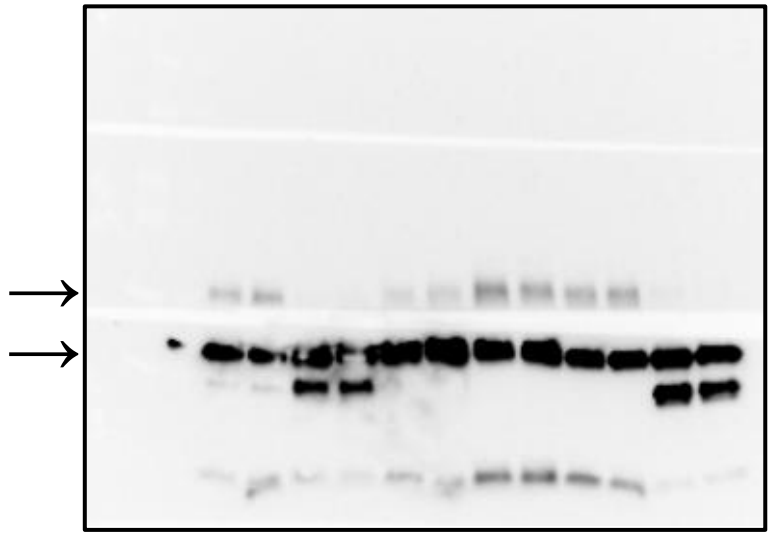

Fig 1C: SM LivARKO Fem 1-Month HFD (AKT, Actin)

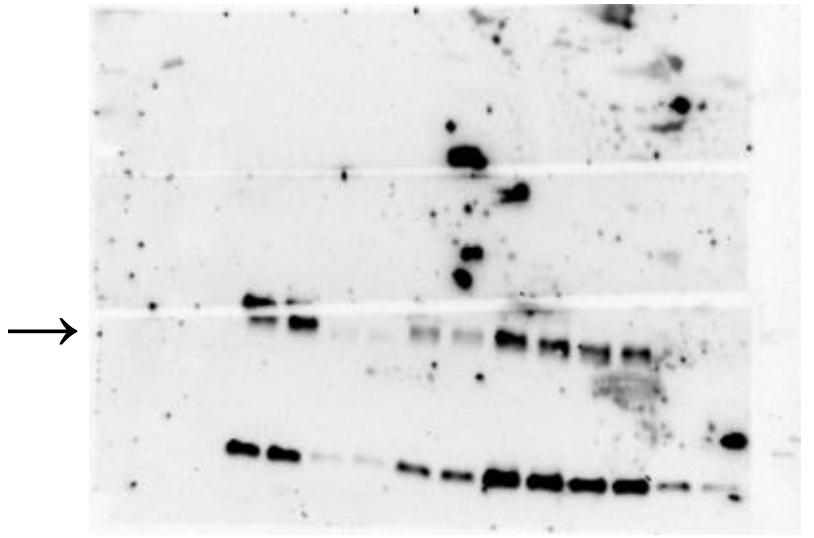

Fig 1C: SM LivARKO Fem 1-Month HFD (p-AKT)

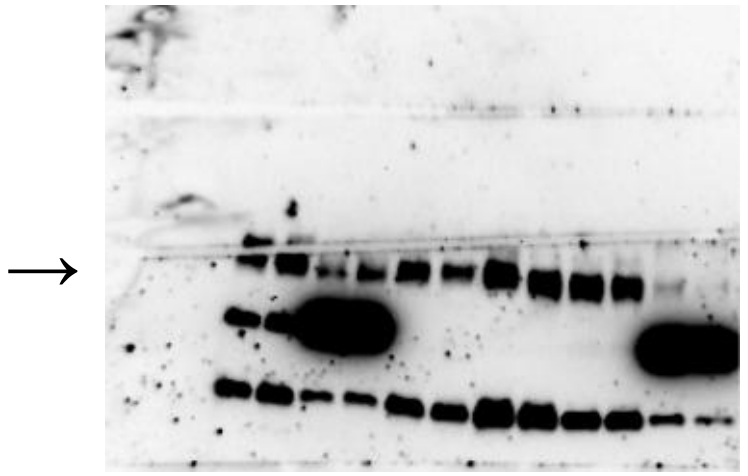

Fig 1C: SM LivARKO Fem 1-Month HFD (AKT)

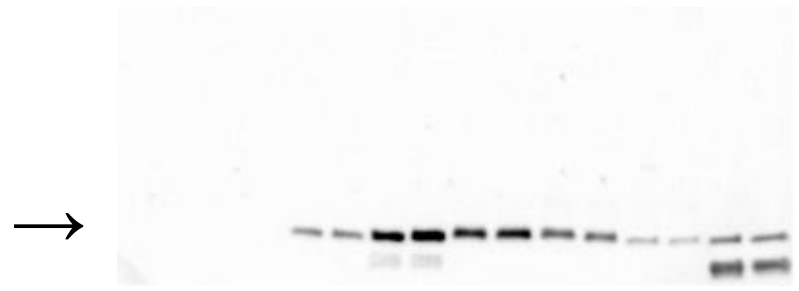

Fig 1C: SM LivARKO Fem 1-Month HFD (Actin)

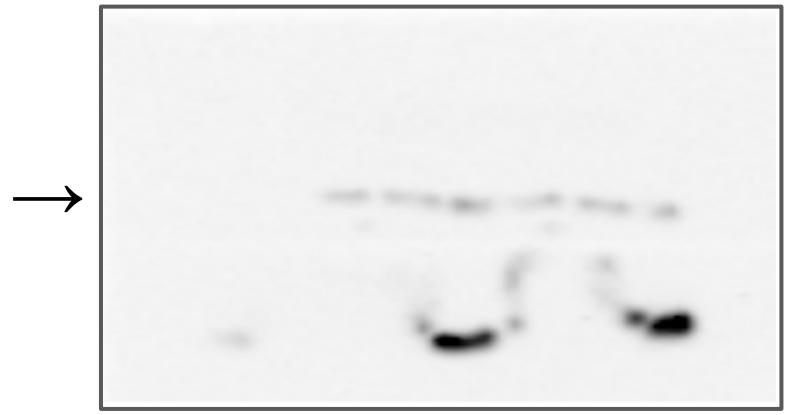

Fig 1E: WAT LivARKO Fem 1-Month HFD (p-AKT)

Green = Analyzed + image used
Yellow = Analyzed but image not used
The best representative image for the figure produced was used.

Some of the nitrocellulose membranes were cut and imaged with the pieces touching so that they look like one, but were probed for different Ab..

In Fig 1, The last (a) or first (b) four lanes were not used in this study.

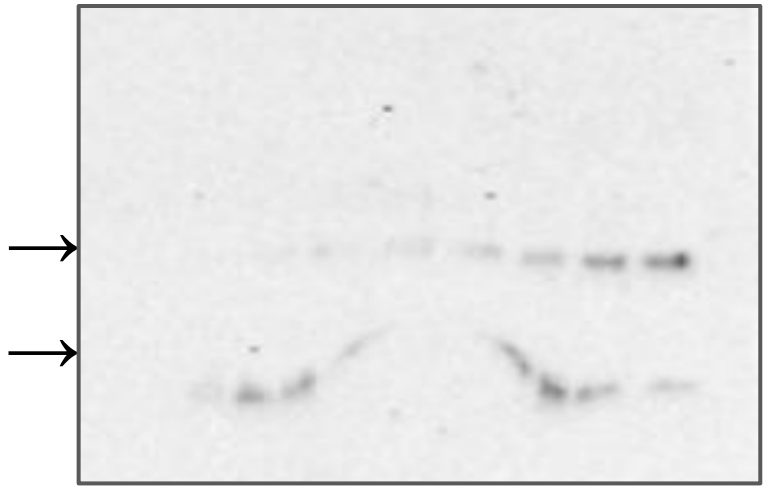

Fig 1E: WAT LivARKO Fem 1-Month (AKT, , GAPDH)

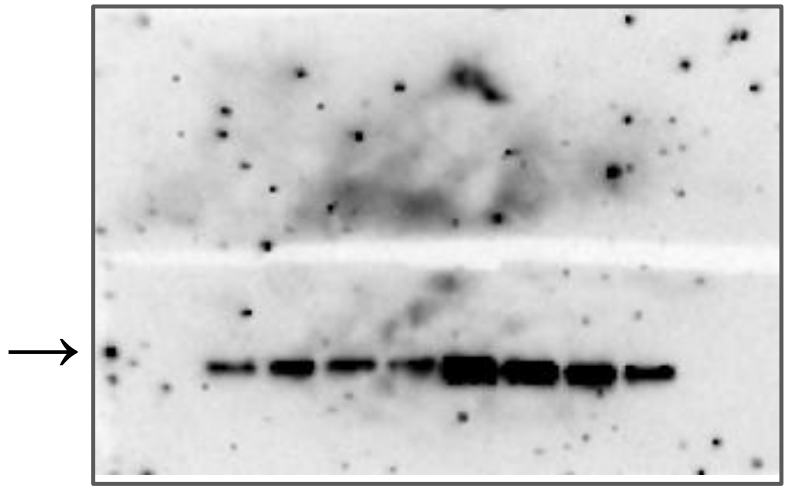

Fig 1E: WAT LivARKO Fem 1-Month (GAPDH)

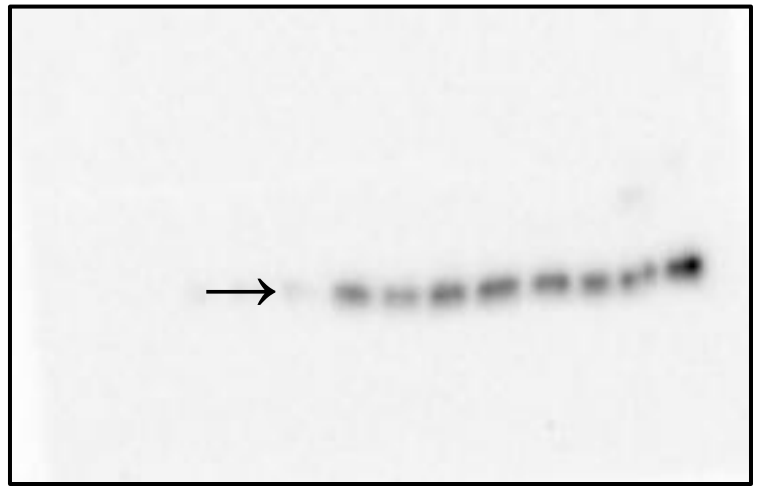

Fig 1E: WAT LivARKO Fem 1-Month (AKT)

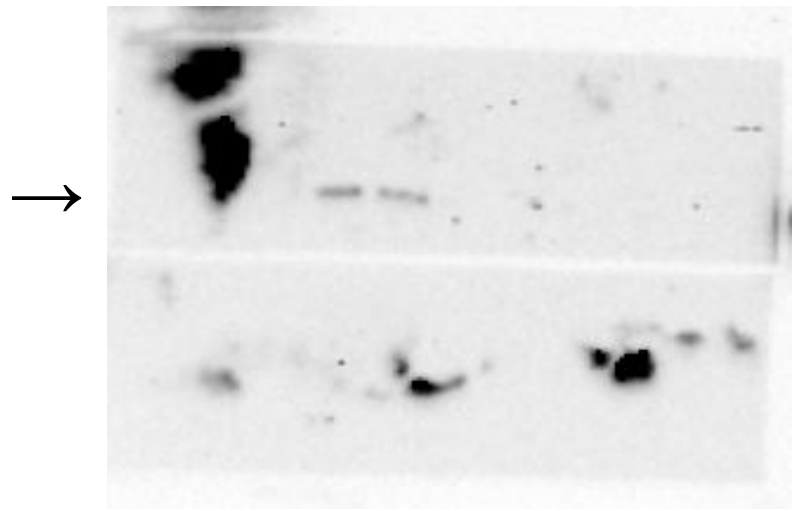

Fig 1E: WAT LivARKO Fem 1-Month HFD (p-AKT)

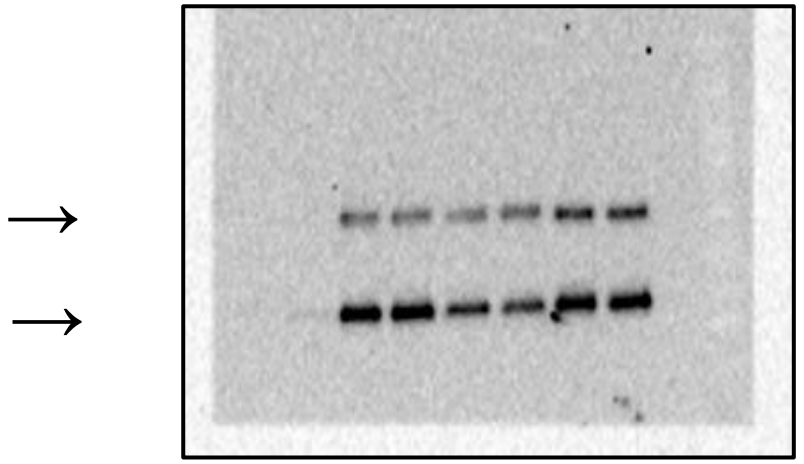

Fig 2A: Liv LivARKO Male 1-Month (p-AKT, GAPDH)

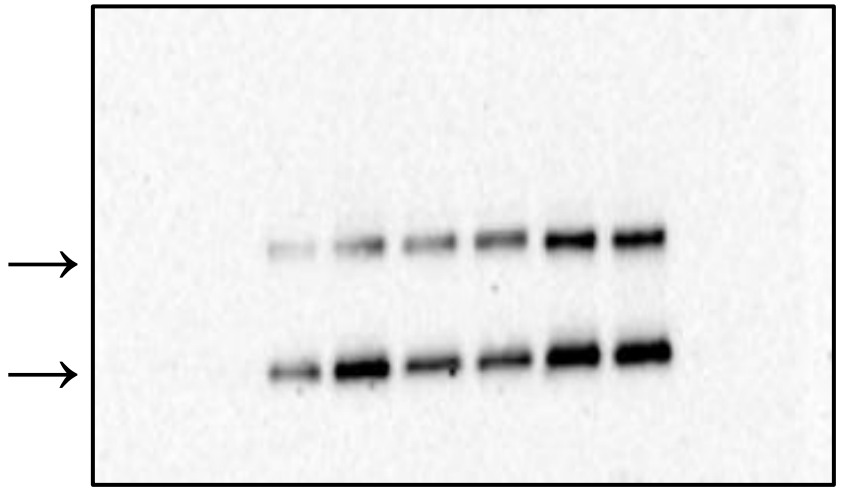

Fig 2A: Liv LivARKO Male 1-Month (AKT, GAPDH)

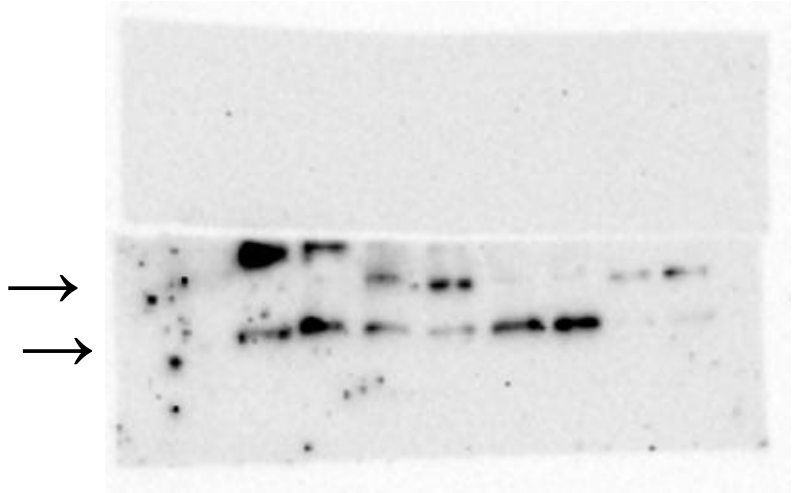

Fig 2A: Liv LivARKO Male 1-Month (p-AKT, Actin)

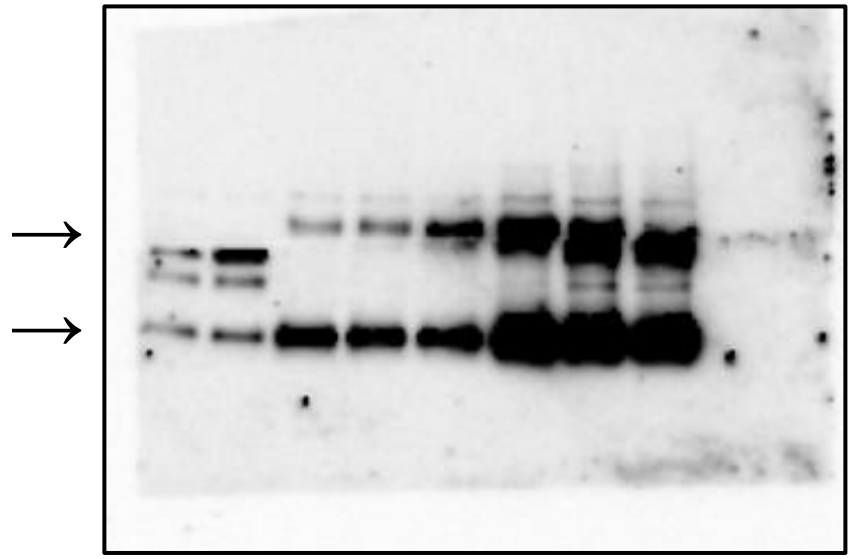

Fig 2A: Liv LivARKO Male 1-Month (AKT, Actin)

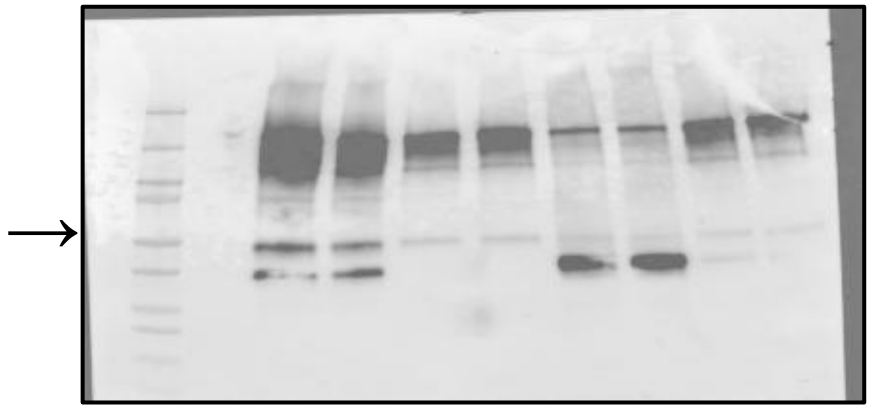

Fig 2E: WAT LivARKO Male 1-Month (p-AKT)

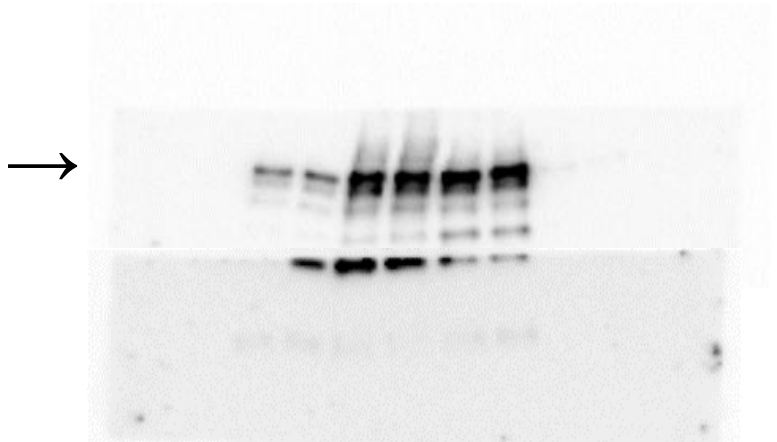

Fig 2E: WAT LivARKO Male 1-Month (p-AKT)

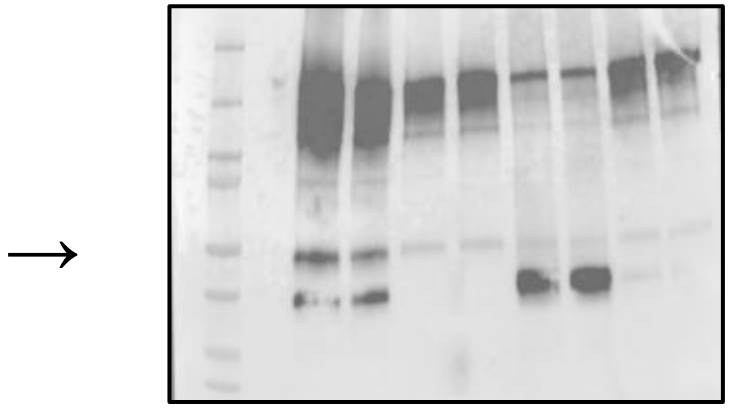

Fig 2E: WAT LivARKO Male 1-Month (AKT)

Green = Analyzed + image used
Yellow = Analyzed but image not used
The best representative image for the figure produced was used.

Some of the nitrocellulose membranes were cut and imaged with the pieces touching so that they look like one, but were probed for different Ab..

In Fig 1, The first four lanes were not used in this study (for some of the blots).

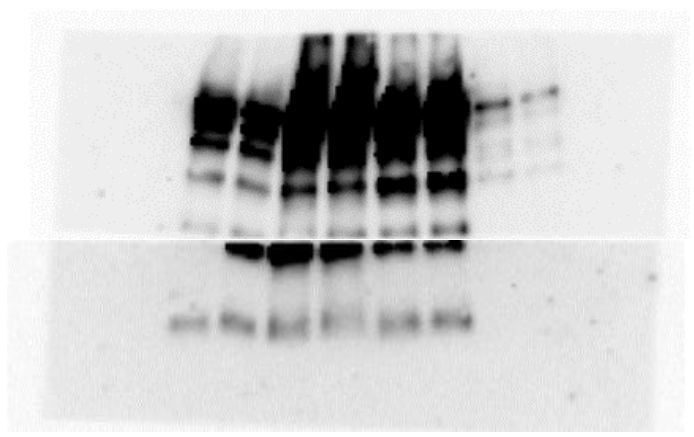

Fig 2E: WAT LivARKO Male 1-Mon HFD (AKT, GAPDH)

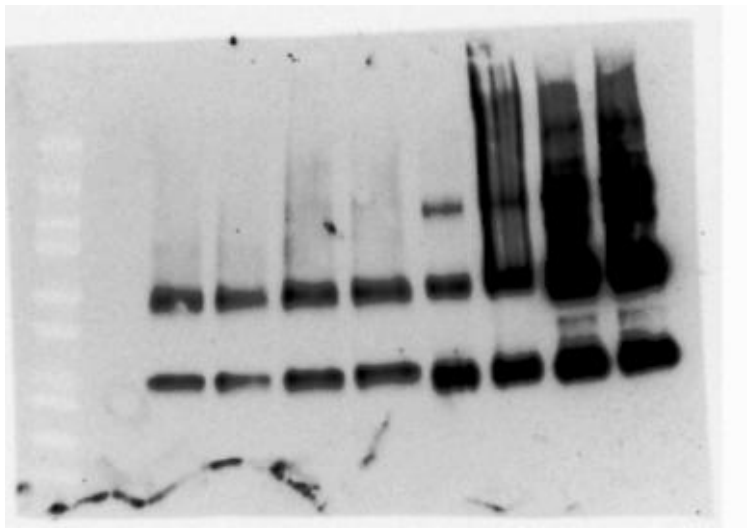

Fig 2E: WAT LivARKO Male 1-Mon HFD (Actin)

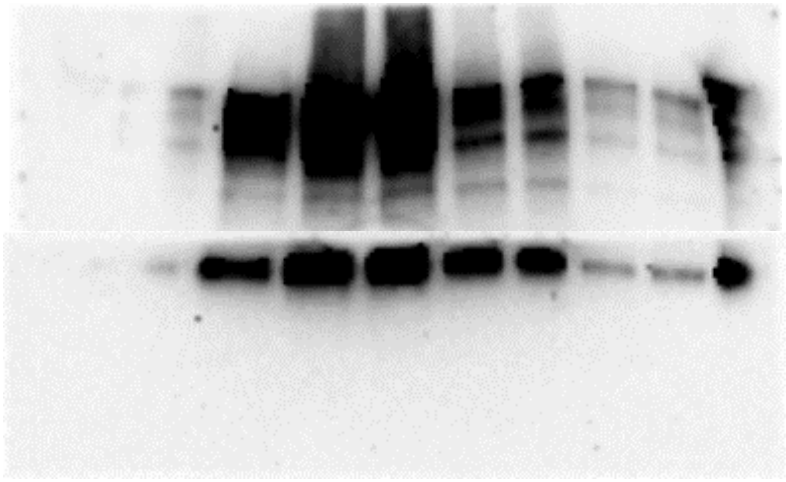

Fig 2E: WAT LivARKO Male 1-Mon HFD (Actin) *different lane order.

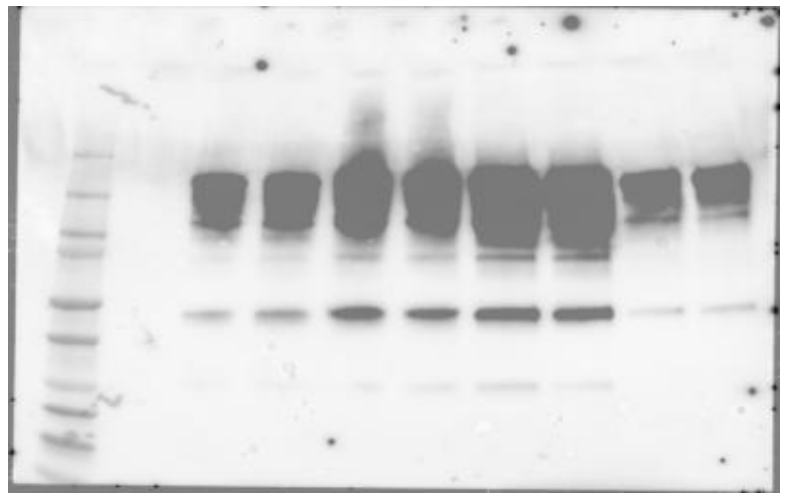

Fig 2C: SM LivARKO Male 1-Mon HFD (AKT)

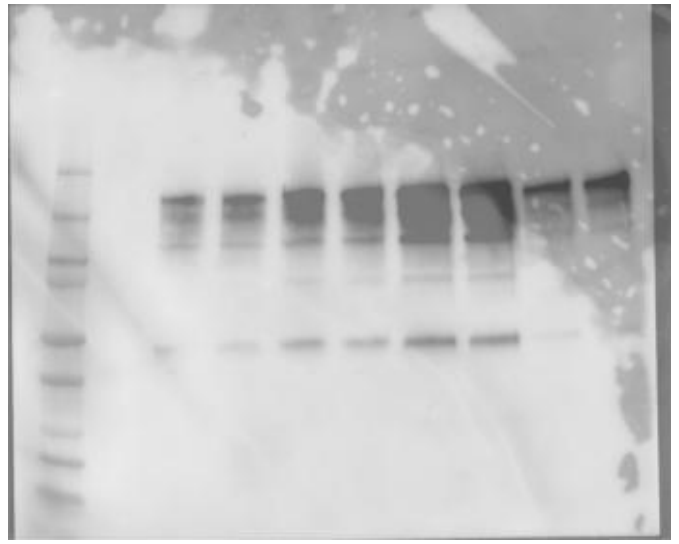

Fig 2C: SM LivARKO Male 1-Mon HFD (p-AKT)

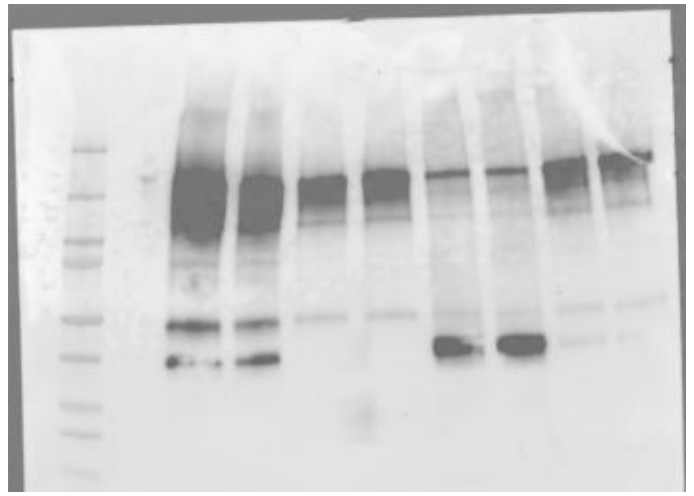

Fig 2C: SM LivARKO Male 1-Mon HFD (Actin)

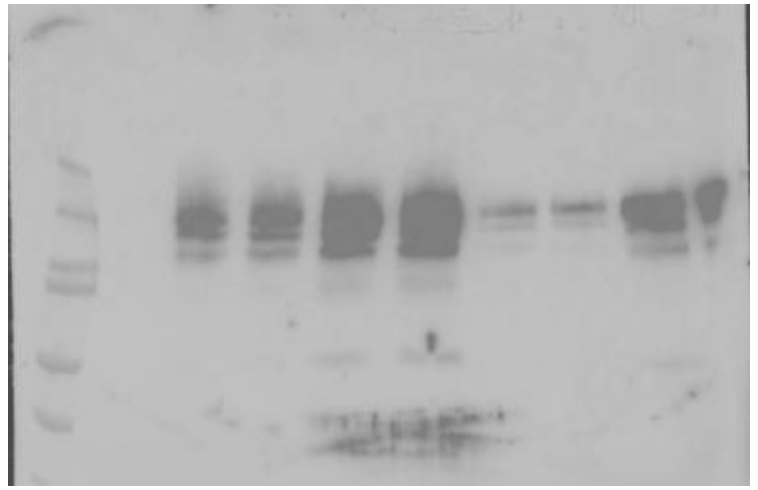

Fig 2C: SM LivARKO Male 1-Mon HFD (p-AKT)

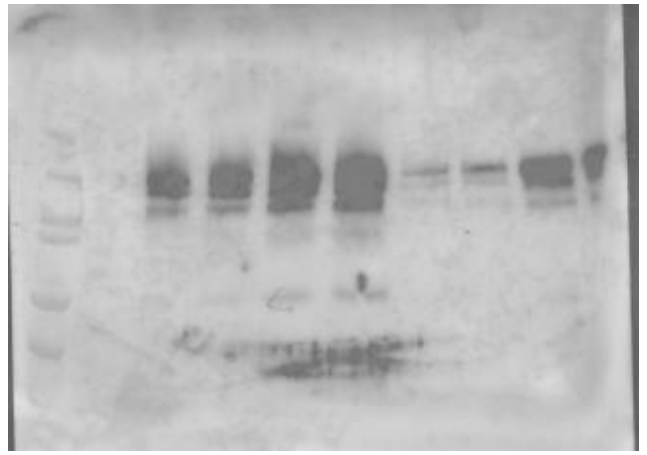

Fig 2C: SM LivARKO Male 1-Mon HFD (AKT)

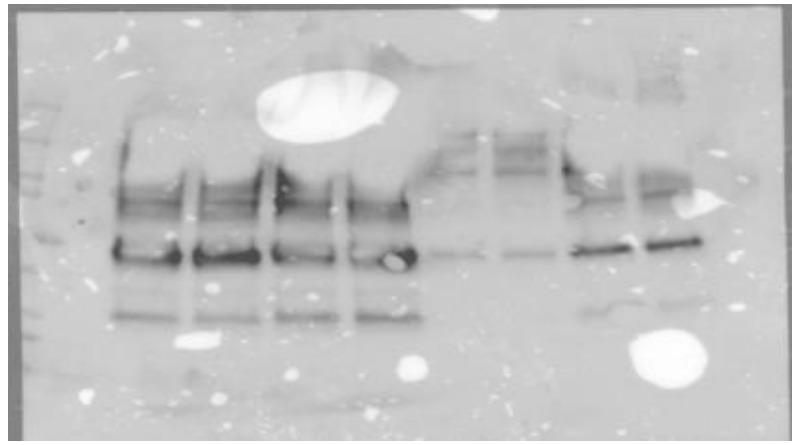

Fig 3A: Liv LivARKO Fem 3-Mon HFD (p-AKT)

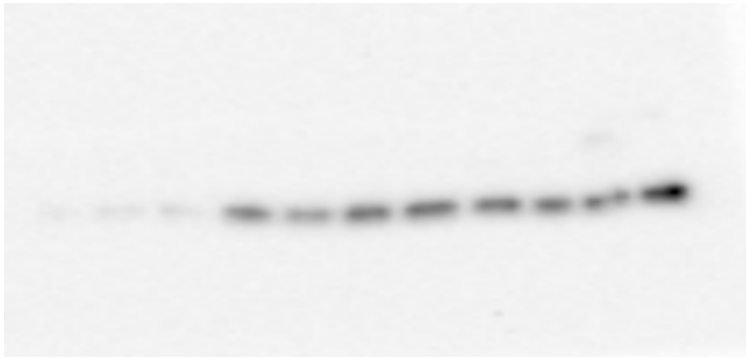

Fig 3A: Liv LivARKO Fem 3-Mon HFD (AKT)

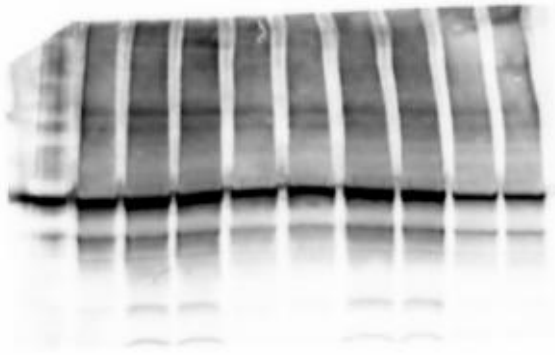

Fig 3A: Liv LivARKO Fem 3-Mon HFD (Actin)

Green = Analyzed + image used
Yellow = Analyzed but image not used
The best representative image for the figure produced was used.

Some of the nitrocellulose membranes were cut and imaged with the pieces touching so that they look like one, but were probed for different Ab..

In Fig 1, The first four lanes were not used in this study.

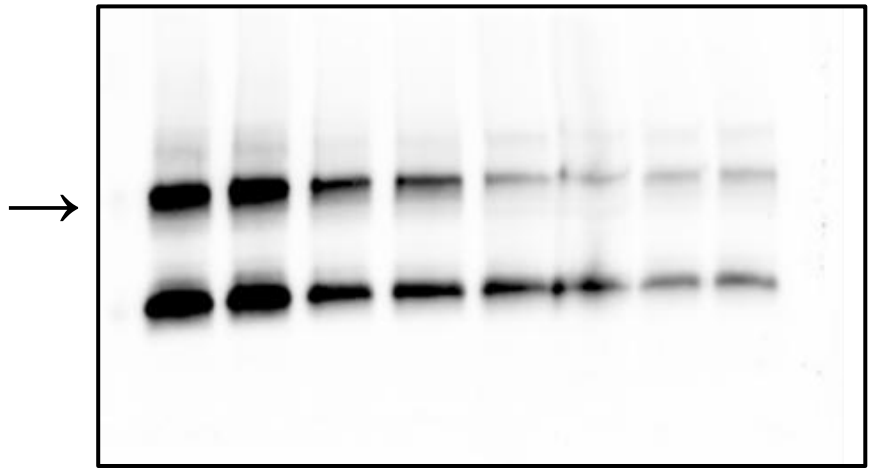

Fig 3C: SM LivARKO Fem 3-Month HFD (p-AKT)

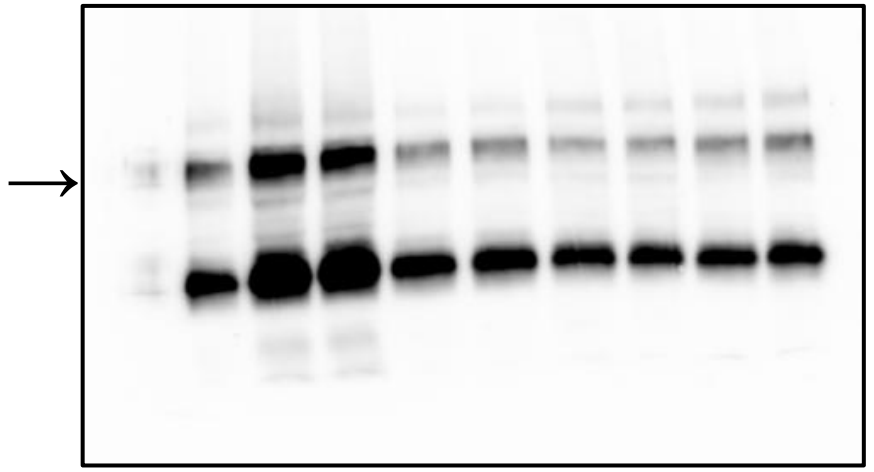

Fig 3C: SM LivARKO Fem 3-Month HFD (AKT)

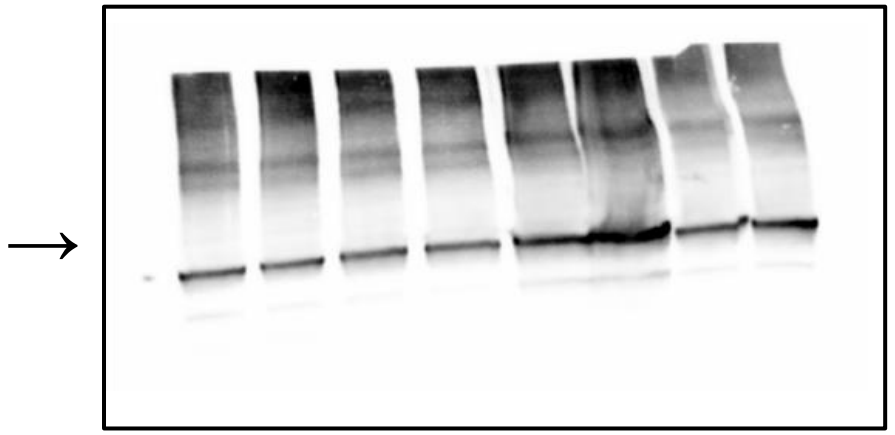

Fig 3C: SM LivARKO Fem 3-Month HFD (Actin)

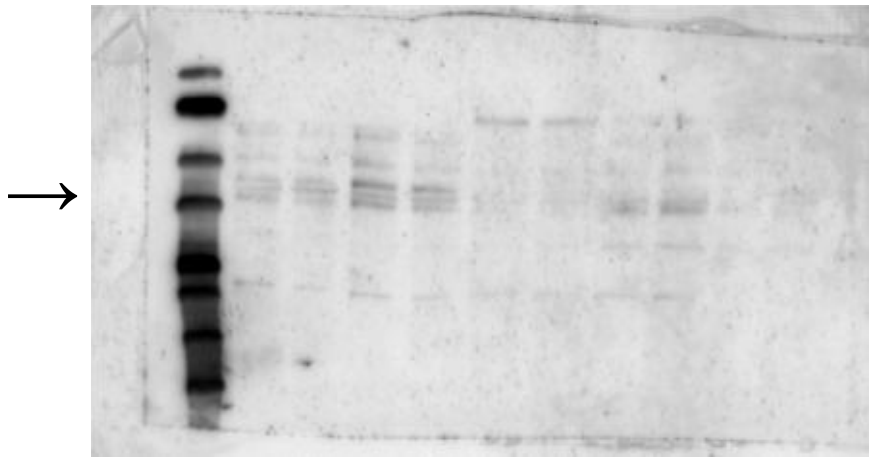

Fig 3E: WAT LivARKO Fem 3-Month HFD (p-AKT)

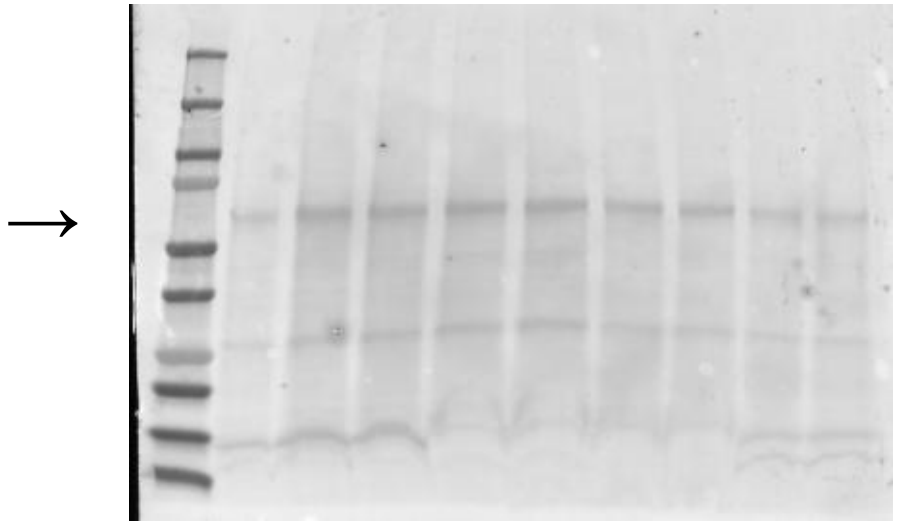

Fig 3E: WAT LivARKO Fem 3-Month HFD (AKT)

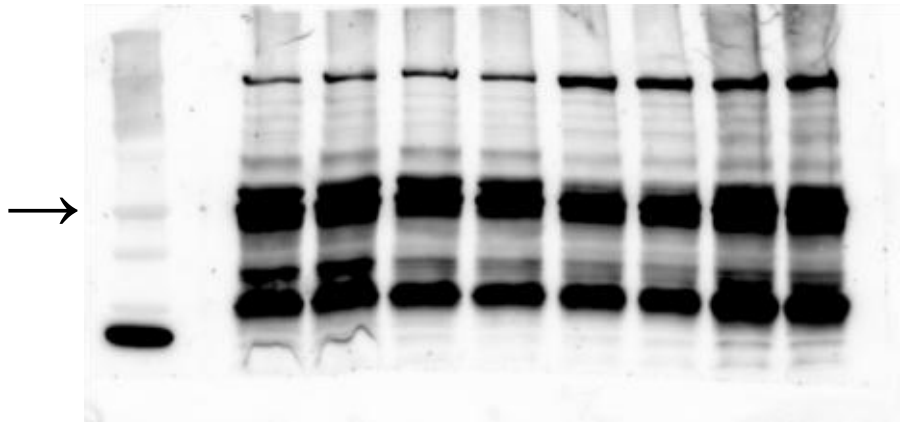

Fig 3E: WAT LivARKO Fem 3-Month HFD (Actin)

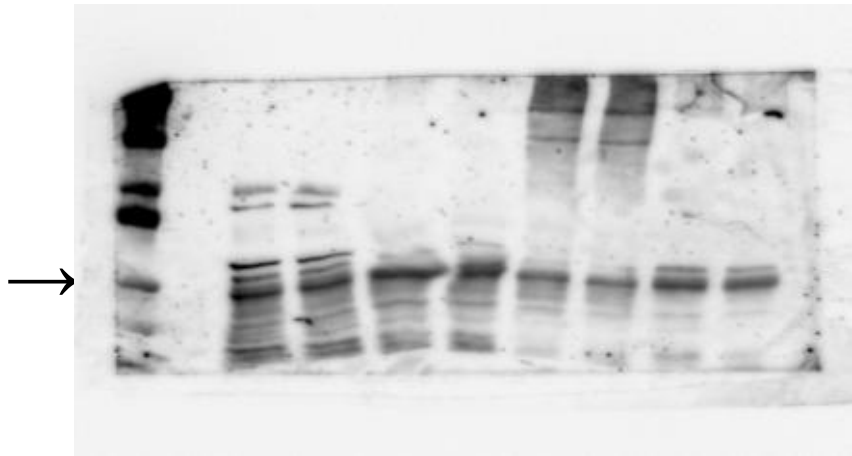

Fig 4A: Liv LivARKO Male 3-Mon HFD (p-AKT)

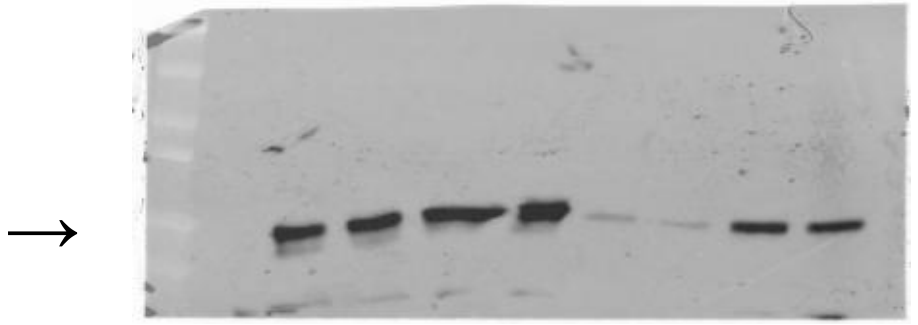

Fig 4A: Liv LivARKO Male 3-Mon HFD (AKT)

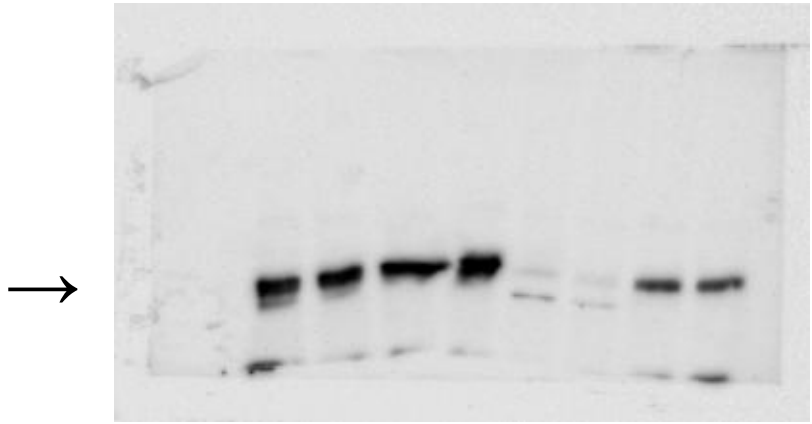

Fig 4A: Liv LivARKO Male 3-Mon HFD (Actin)

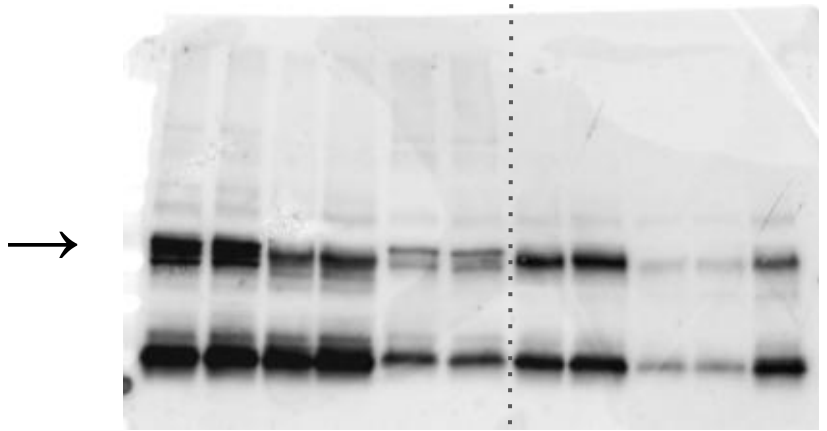

Fig 4A: Liv LivARKO Male 3-Mon HFD (p-AKT)

Fig 4C: SM LivARKO Male 3-Mon HFD (p-AKT)

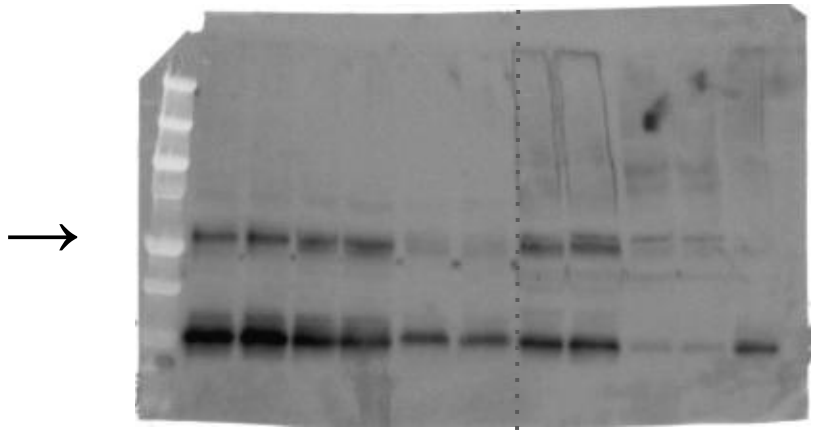

Fig 4A: Liv LivARKO Male 3-Mon HFD (AKT)

Fig 4C: SM LivARKO Male 3-Mon HFD (AKT)

Green = Analyzed + image used
Yellow = Analyzed but image not used
The best representative image for the figure produced was used.

Some of the nitrocellulose membranes were cut and imaged with the pieces touching so that they look like one, but were probed for different Ab..

Fig 4A Lanes: Con Ins, Con Bas, HFD Ins
Fig 4C Lanes: Con Bas, HFD Ins, Con Ins

→

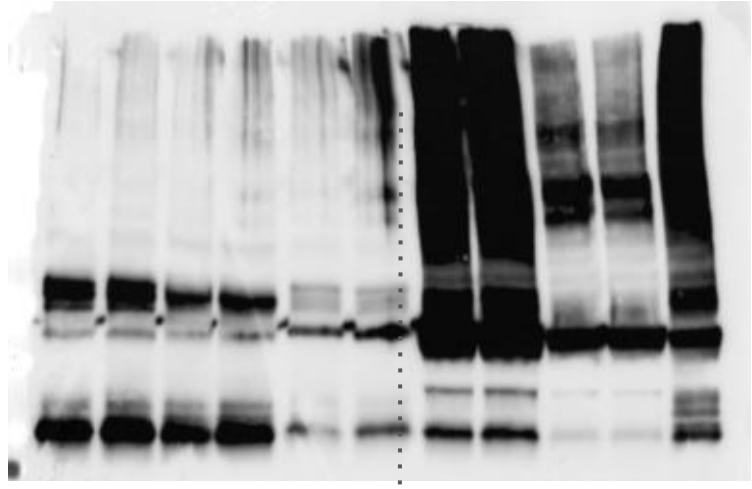

Fig 4A: Liv LivARKO Male 3-Mon HFD (Actin)

Fig 4C: SM LivARKO Male 3-Mon HFD (Actin)

→

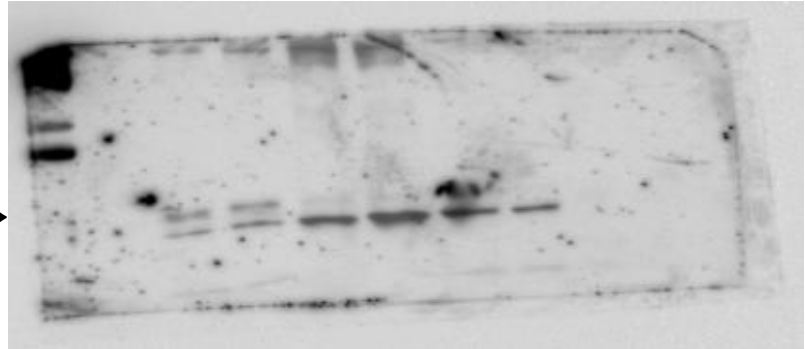

Fig 4C: SM LivARKO Male 3-Month HFD (p-AKT)

→

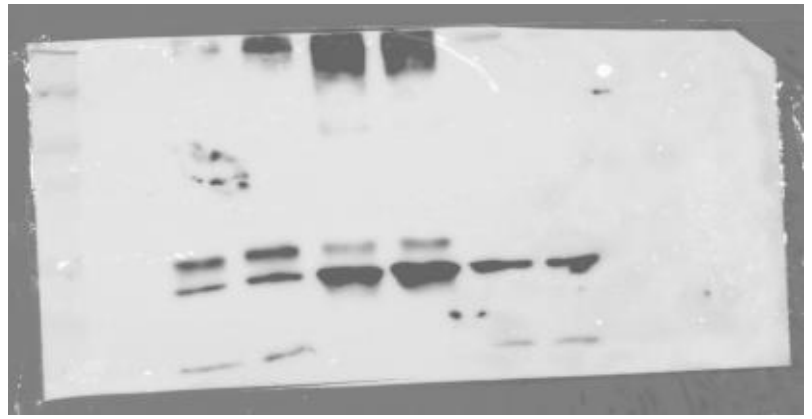

Fig 4C: SM LivARKO Male 3-Month HFD (AKT)

→

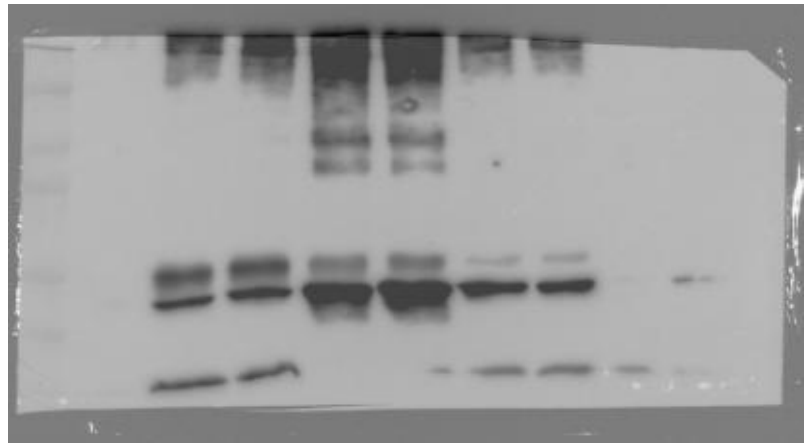

Fig 4C: SM LivARKO Male 3-Month HFD (Actin)

→

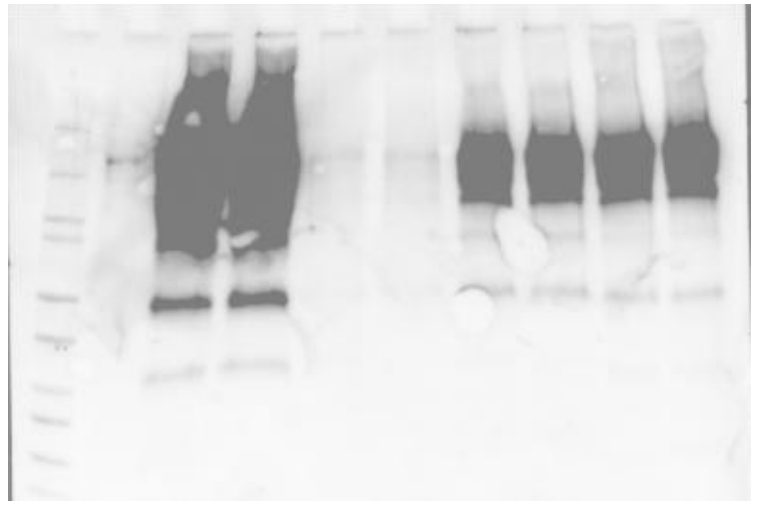

Fig 4E: WAT LivARKO Male 3-Month HFD (p-AKT)

→

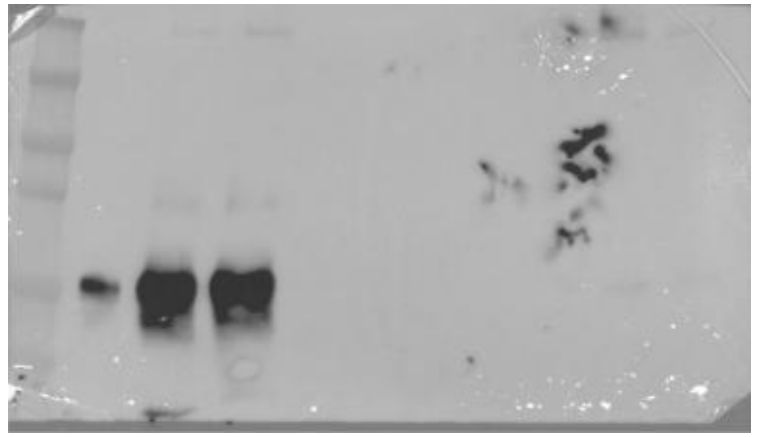

Fig 4E: WAT LivARKO Male 3-Month HFD (AKT)

→

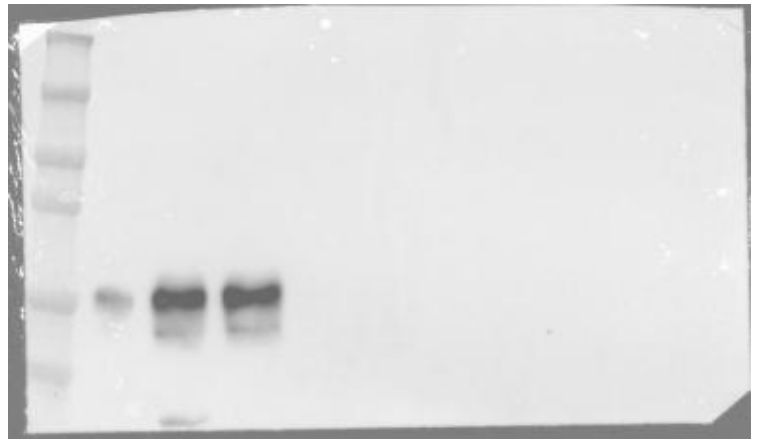

Fig 4E: WAT LivARKO Male 3-Month HFD (Actin)

→

→

→

→

→

Green = Analyzed + image used
Yellow = Analyzed but image not used
The best representative image for the figure produced was used.

Some of the nitrocellulose membranes were cut and imaged with the pieces touching so that they look like one, but were probed for different Ab..

In Fig 1, The first four lanes were not used in this study.

Supplemental Figure 1. Validation of liver-specific androgen receptor deletion in LivARKO mice.

Relative androgen receptor (*Ar*) mRNA expression was measured by quantitative RT-PCR in liver, skeletal muscle (SKM), and white adipose tissue (WAT) from wild-type (WT) and liver-specific androgen receptor knockout (LivARKO) mice. *Ar* mRNA levels were significantly reduced in liver tissue from LivARKO mice compared with WT controls, confirming efficient hepatic deletion of *Ar*. In contrast, *Ar* expression in SKM and WAT were unchanged between genotypes, demonstrating tissue specificity of the knockout. RNA was isolated from frozen tissues, reverse transcribed, and analyzed by SYBR Green qPCR. Gene expression was normalized to *Actb* using the ΔΔCt method. Data are presented as mean ± SEM; $n = 8$ biological replicates per group

Supplemental Figure 2. High-fat diet impairs insulin-stimulated Akt phosphorylation in metabolic tissues of male mice.

Phosphorylated Akt (Ser473) and total Akt levels were quantified by sandwich ELISA in liver (A), skeletal muscle (B), and white adipose tissue (WAT) (C) from male wild-type (WT) mice maintained on either a control diet or high-fat diet (HFD). Mice were fasted for 6 h prior to tissue collection, and insulin-stimulated groups received insulin (0.5 U/kg body weight, intraperitoneal injection) 10 min before sacrifice. Basal WT control values (WT-Con-Bas) were normalized to 1.0 for each tissue. Acute insulin stimulation robustly increased Akt phosphorylation in liver, skeletal muscle, and WAT of control diet-fed mice. In contrast, HFD feeding markedly blunted insulin-stimulated Akt phosphorylation across all tissues. Protein lysates were prepared from snap-frozen tissues, and phospho-Akt values were normalized to corresponding total Akt measurements. Data are presented as mean ± SEM; $n = 3$ biological replicates per group.

Supplemental Figure 3. High-fat diet suppresses insulin-stimulated Akt phosphorylation in metabolic tissues of female mice.

Phosphorylated Akt (Ser473) and total Akt levels were quantified by sandwich ELISA in liver (A), skeletal muscle (B), and white adipose tissue (WAT) (C) from female wild-type (WT) mice maintained on either a control diet or high-fat diet (HFD). Mice were fasted for 6 h prior to tissue collection, and insulin-stimulated groups received insulin (0.5 U/kg body weight, intraperitoneal injection) 10 min before sacrifice. Basal WT control values (WT-Con-Bas) were normalized to 1.0 for each tissue. Insulin robustly stimulated Akt phosphorylation in all tissues from control diet-fed mice, whereas HFD feeding substantially reduced insulin-induced Akt activation in liver, skeletal muscle, and WAT. These findings demonstrate impaired insulin signaling at the level of Akt phosphorylation in female HFD-fed mice. Protein lysates were prepared from snap-frozen tissues, and phospho-Akt values were normalized to corresponding total Akt measurements. Data are presented as mean ± SEM; $n = 3$ biological replicates per group.

Supplementary Figure 4. Uncropped, unprocessed immunoblot images corresponding to Figures 1–4.

Uncropped, unprocessed scans of all Western blots used for the analyses presented in Figures 1–4 are shown. Images were acquired without adjustments to contrast, brightness, gamma, or other nonlinear image processing. Molecular weight markers are included on the original scans. Green boxes indicate the representative blot images that were used for quantitative analysis and included in the main figures. Yellow boxes indicate blots that were quantified but from which an alternative representative image was selected for presentation. When multiple technically equivalent blots were available, the image providing the clearest representative visualization of the quantified data was used in the main figures. Where nitrocellulose membranes were cut after

protein transfer to permit probing with different primary antibodies, the membrane sections are displayed adjacent to one another in this supplementary figure for ease of visualization. These membrane sections were probed independently and do not represent a continuous membrane. No digitally spliced images were used to create a single continuous blot in the main figures. For the cropped blots presented in the main figures, only lanes corresponding to the experimental groups included in the study are shown. Omitted lanes are indicated in the uncropped scans and were separated from the displayed lanes by visible dividing spaces in the published figures.
Specifically:

- **Figure 1:** For selected blots, the first four lanes or the last four lanes contained samples not included in this study and were excluded from the final figure.
- **Figure 2:** For selected blots, the first four lanes contained samples not included in this study and were excluded from the final figure.
- **Figure 4:** The order of lanes differed between liver and skeletal muscle blots because samples were run on separate gels. The lane identities are indicated on the uncropped images (e.g., Control Basal, Control Insulin, and HFD Insulin).

Loading controls (β-actin or GAPDH) correspond to the same samples analyzed for the associated target protein. In cases where loading controls and target proteins were obtained from separate membrane sections or independently probed membrane fragments, this is indicated on the uncropped images. All cropping retained sufficient background above and below the protein bands, and all quantitative analyses were performed using the uncropped original images prior to preparation of the representative figures.

ARRIVE Item	Section/Line (or Response)
**1. Study design**	**Methods – Experimental Design; Animal Ethics, Husbandry, Randomization, and Experimental Procedures.** Experimental groups consisted of WT and LivARKO male and female mice fed control or HFD diets, with basal and insulin-stimulated conditions. Experimental unit = individual mouse.
**2a. Sample size**	**Figure Legends; ELISA Methods; Experimental Design.** Western blot experiments: n=2–4 depending on tissue/timepoint. ELISA/qPCR: n=3 biological replicates. Sample sizes are reported in figure legends.
**2b. Sample size calculation**	**Methods – Power Analysis.** Resource equation method with consultation from a biostatistician. No formal a priori power calculation was performed.
**3a. Inclusion/exclusion criteria**	**Methods - Animal Ethics, Husbandry, Randomization, and Experimental Procedures.** No predefined inclusion or exclusion criteria were established before the study. All animals completing the experimental protocol were included in the analyses.
**3b. Exclusions**	**Discussion.** Stated that no animals were excluded from analysis. Mentioned that Figure 4E represented technical variability rather than exclusion of animals.
**3c. Exact n**	**Figure Legends**
**4a. Randomization**	**Animal Ethics section.** Animals were randomly assigned to diet groups.
**4b. Confounders**	**Methods - Animal Ethics, Husbandry, Randomization, and Experimental Procedures:** Animals from different experimental groups were housed under identical environmental conditions, and tissue collection and molecular analyses were performed using standardized protocols to minimize batch effects.
**5. Blinding**	**Animal Ethics section.** Investigators performing quantitative image analysis were blinded during analysis.
**6a. Outcome measures**	**Results; Methods.** Primary outcomes include p-AKT (Ser473), total AKT, Pck1, G6pc, and Ar mRNA.
**6b. Primary outcome**	**Protein Processing and Western Blots.** The primary outcome measure was insulin-stimulated hepatic AKT phosphorylation.

**7a. Statistical methods**	**Statistical Analysis.** Two-way ANOVA, Pearson correlation, Fisher's exact test, Prism software.
**7b. Assumptions**	**Statistical Analysis.** Add: "Data were assessed for normality and homogeneity of variance before parametric analyses. No violations requiring alternative statistical methods were identified."
**8a. Experimental animals**	**Generation of LivARKO Mice; Experimental Design.** Species, strain, sex, genotype, age.
**8b. Provenance**	**Generation of LivARKO Mice.** Jackson Laboratory source, genetic modification described.
**9. Experimental procedures**	**Methods throughout.** Generation of mice, insulin injections, qPCR, Western blotting, ELISA, diet composition, fasting, tissue harvest.
**10a. Results**	**Results and Figure Legends.** Means ± SEM reported.
**10b. Effect size**	**Not reported.** Not calculated.

ARRIVE Item	Response
**11 Abstract**	Fully addressed.
**12 Background**	Introduction.
**13 Objectives**	End of Introduction.
**14 Ethical statement**	Animal Ethics section (Howard IACUC).
**15 Housing/Husbandry**	Animal Ethics section.
**16 Animal care & monitoring**	**Methods - Animal Ethics, Husbandry, Randomization, and Experimental Procedures.** Animals were monitored daily for health and welfare by trained personnel. No unexpected adverse events occurred, and no humane endpoints were reached before the planned study endpoint.
**17 Interpretation**	Discussion.
**18 Generalizability**	Discussion.

**19 Protocol registration**	No protocol was prospectively registered for this study.<br>Section: Protocol Registration.
**20 Data access**	Data Availability statement.
**21a Conflicts**	Competing Interests statement.
**21b Funding**	Acknowledgements.
